# Keep out! SARS-CoV-2 entry inhibitors: their role and utility as COVID-19 therapeutics

**DOI:** 10.1186/s12985-021-01624-x

**Published:** 2021-07-23

**Authors:** Lennox Chitsike, Penelope Duerksen-Hughes

**Affiliations:** Department of Basic Sciences, Loma Linda University School of Medicine, 11021 Campus Street, 101 Alumni Hall, Loma Linda, CA 92354 USA

**Keywords:** SARS-CoV-2, Covid-19, Viral entry inhibitors, Antibodies, SARS-CoV-2 variants, Prophylaxis, Emerging sarbecoviruses

## Abstract

The COVID-19 pandemic has put healthcare infrastructures and our social and economic lives under unprecedented strain. Effective solutions are needed to end the pandemic while significantly lessening its further impact on mortality and social and economic life. Effective and widely-available vaccines have appropriately long been seen as the best way to end the pandemic. Indeed, the current availability of several effective vaccines are already making a significant progress towards achieving that goal. Nevertheless, concerns have risen due to new SARS-CoV-2 variants that harbor mutations against which current vaccines are less effective. Furthermore, some individuals are unwilling or unable to take the vaccine. As health officials across the globe scramble to vaccinate their populations to reach herd immunity, the challenges noted above indicate that COVID-19 therapeutics are still needed to work alongside the vaccines. Here we describe the impact that neutralizing antibodies have had on those with early or mild COVID-19, and what their approval for early management of COVID-19 means for other viral entry inhibitors that have a similar mechanism of action. Importantly, we also highlight studies that show that therapeutic strategies involving various viral entry inhibitors such as multivalent antibodies, recombinant ACE2 and miniproteins can be effective not only for pre-exposure prophylaxis, but also in protecting against SARS-CoV-2 antigenic drift and future zoonotic sarbecoviruses.

## Introduction

COVID-19, the disease caused by the novel coronavirus SARS-CoV-2, was declared a pandemic and global emergency shortly after it began in late 2019. As of today, the disease has claimed about 3 million lives and cost the world trillions of dollars [1, 2]. Even though the majority of people recover from the disease and experience only mild or no symptoms, the pathogenicity and transmissibility of the virus grants COVID-19 a higher burden of mortality than seen in other ongoing viral diseases such as the seasonal flu caused by influenza [3, 4]. Given this fatality rate and the novelty of the virus, the science surrounding COVID-19 has been a rapidly evolving field. Developing and validating treatment strategies has required a delicate balance between rigor in scientific evaluation and expediency in developing therapies that help to slow down the rampage of the pandemic. The scientific community continues to unravel the nature of SARS-CoV-2, though we now know much more about SARS-CoV-2 and the consequences of infection than when it was first discovered [5–7].

**Fig. 1:**
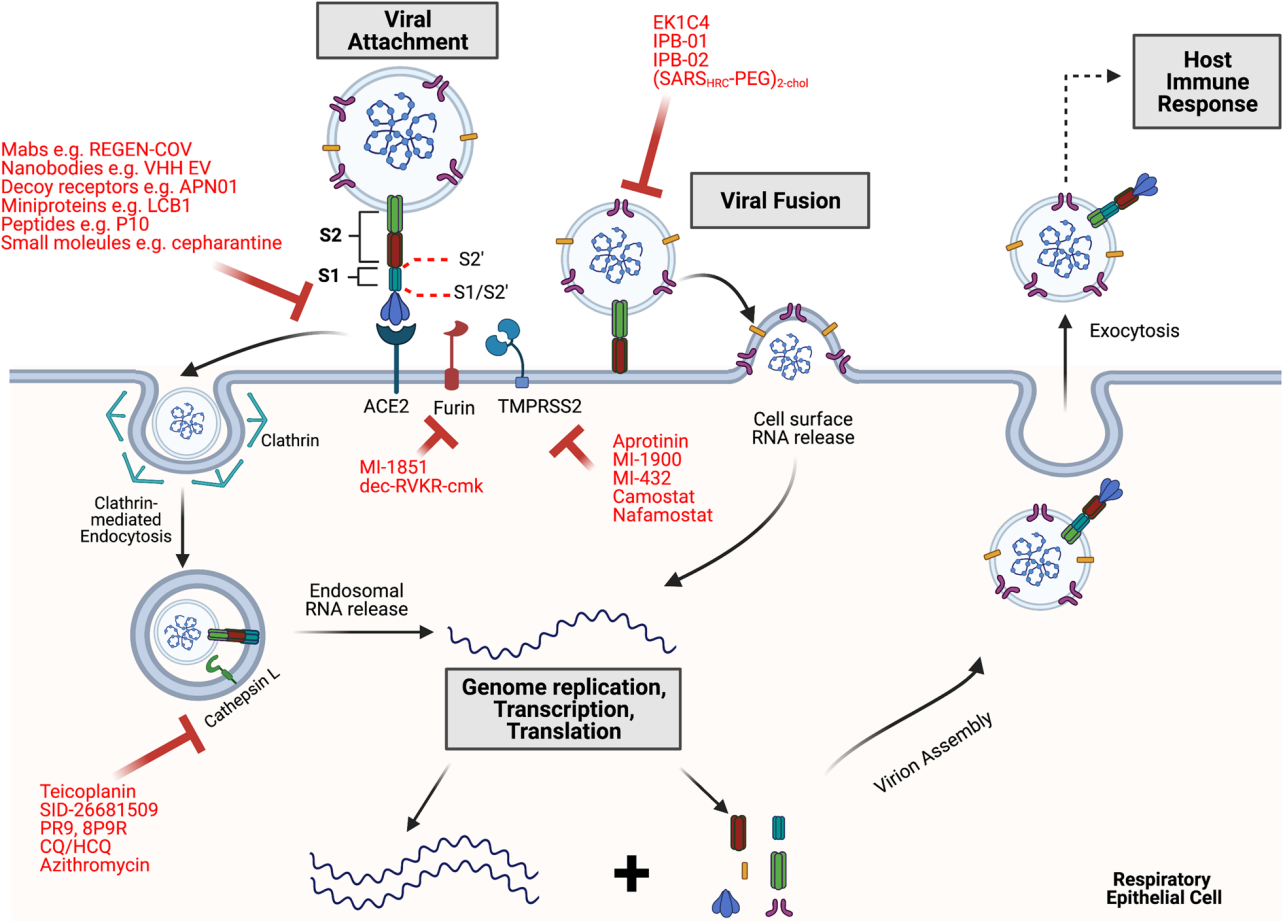
Overview of SARS-CoV-2 life cycle and inhibitors of viral entry. SARS-CoV-2 first binds to ACE2 on the cell surface and then releases RNA into the cytosol from the endosome following endocytosis and cathepsin activation, or directly from the cell surface membrane following S activation by proteases such as furin and TMPRSS2. In the cytosol, the ( +) genomic RNA is directly translated from the ORF1a/b into polyproteins containing non-structural proteins of the complex replicase machinery (e.g. RdRp). (-) sense RNA is synthesized and becomes a template for ( +) sense genomic RNA and sub-genomic RNA from which structural proteins and accessory proteins are made. These proteins and the genomic RNA will be utilized to assemble new virions that will exit the cell via exocytosis. The virions and the death of infected cell will induce immune response from the host. At each stage of entry shown, a number of candidates (shown in red) that inhibit each of the highlighted viral entry checkpoints (including cellular receptors, enzymes and viral proteins) have demonstrated efficacy against infection, preclinically, clinically or both. (Figure created using Biorender.)

SARS-CoV-2 is an enveloped, positive-sense, single-stranded RNA from the betacoronavirus genus [6, 7]. The major entry point is the nasal passage, from which infection begins following exposure [8–10]. Specifically, the virus enters the nasal epithelial cells through the binding of the viral Spike (S) glycoprotein to its cellular receptor known as angiotensin-converting enzyme-2 (ACE2). Following binding, the virus will gain access into the cell via endocytosis or fusion with surface cell membrane. After the virus has unloaded its genome into the cytoplasm, it hijacks the host translational machinery and directs the production of large polyproteins from which essential proteins such as RNA dependent RNA polymerase and helicase are made via viral proteolytic cleavage. The replication proteins will generate genomic RNA as well as sub-genomic RNA that become templates for the synthesis of accessory and structural proteins [3, 7, 11]. The genomic RNA and the proteins are then assembled into virions that exit the cell via exocytosis and infect new cells (Fig. 1). Later on, the virus may spread to the lower respiratory system through aspiration and/or infection of cells in conducting airways [3, 7–10]. Through a timely and balanced immune response involving the innate and adaptive response, viral propagation is managed and mostly confined to upper airways, resulting in mild symptoms being observed in the majority of patients [12, 13].

However, some individuals will go on to develop more severe symptoms, likely due to successful immune evasion by the virus and/or delayed and impaired responses by their immune systems [9, 12, 13]. It has been shown that a balanced response involving innate immunity, B cells, CD4^+^ T cells, and CD8^+^ T cells is needed to control SARS-CoV-2 [12]. In the absence of this balanced response, the virus will eventually reach the pulmonary gas exchange units and infect type II alveolar cells [9]. A dampened initial immune response will allow the virus to replicate and spread to new cells. The infected cells will undergo apoptosis and die, with their death leading not only to alveolar damage but also an excessive production of pro-inflammatory cytokines such as IL-6, IFN-γ, IL-8, TNF-α, and IL-1β [3, 9, 11, 14, 15]. The release of these cytokines by host cells (also known as cytokine storm) will further distort the antiviral immune response and usually coincides with recruitment of immune cells such as monocytes and neutrophils [12, 16]. An interplay of these events may lead to a vicious cycle in severe cases, marked by complications such as increased vascular permeability, pulmonary edema, acute respiratory distress syndrome (ARDS), multi-organ failure and death [3, 11, 15].

These emerging concepts regarding SARS-CoV-2 and the pattern of clinical progression of COVID-19 have clarified several issues. For example, we now know that there are two phases in the pathogenesis of COVID-19, and that treatment of the disease requires two distinct strategies. Specifically, in the early phase of COVID-19, viral growth and propagation are the primary determinants driving disease progression or resolution. In the later phases, a hyperinflammatory response by the host is much more important in driving the disease than is viral replication [11, 17]. This understanding has now translated into current approaches to treat COVID-19. Treatment for outpatients diagnosed early with mild to moderate COVID-19, but who are at risk of hospitalization due to comorbidities or other factors, often involves administration of neutralizing anti-SARS-CoV-2 monoclonal antibodies, as discussed below. However, patients that are hospitalized may be given redemsevir, dexamathosone, bariticinib or a combinatorial regimen comprising these, depending on whether the patient requires supplemental oxygen and ventilation [18]. Importantly, targeting the virus during the early phase of infection means that significant benefit can be gained from rapid viral testing, as a quick diagnosis can capture a therapeutic window of opportunity before an exuberant host-mediated immune response leads to potentially fatal complications such as pneumonia, ARDS, multi-organ system dysfunction and hypercoagulation [19]. Such early treatment of COVID-19 can shorten disease recovery rates, prevent hospitalizations and be a more cost-effective way to manage COVID-19 [11, 17]. Thus far, monoclonal antibodies have been approved for early management of COVID-19. Studies have shown significant neutralization potency and efficacy, providing proof of principle that targeting viral entry can be an effective way to treat COVID-19, at least in the early stages [18, 20]. These encouraging results have not, however, adequately addressed the potential of other types of entry inhibitors. As the mission of a vaccine-driven end to this pandemic faces new challenges due to the rise of mutant variants, new questions are emerging about how these other potential therapeutics may help to alleviate these problems.

In this review we summarize the development of anti-SARS-CoV-2 neutralizing monoclonal antibodies for clinical use and the impact they have had on early management of mild-to-moderate COVID-19. These concepts are discussed in the context of the emerging evidence showing that currently available vaccines are challenged by the rise of new SARS-CoV-2 variants. We also highlight the challenges that antibodies are facing as they deal with SARS-CoV-2 mutants and how, together with a multitude of other emerging candidates for entry inhibition, they can overcome those challenges. Finally, we discuss and evaluate the potential of entry inhibitors as prophylactic agents, as well as the role they can play against emerging SARS-CoV-2 lineages and future coronavirus outbreaks.

## Viral entry inhibitors and their translational relevance

The availability of several effective vaccines against SARS-CoV-2 has given hope to billions of people across the globe [21, 22]. Since the pandemic started, vaccination has appropriately been viewed as the long term and most sustainable solution to deal with COVID-19. It is therefore encouraging that currently available vaccines have been shown to induce both humoral and cellular immunity and to provide substantial protection to vaccinees in clinical trials [12, 23]. Simultaneous with the continued rollout of vaccines across the globe, more and more people will also acquire protective immunity due to infection and recovery from the disease. Nevertheless, it is critical to note that the availability of the vaccine does not obviate the need for available therapies nor for the development of new and more effective therapies. First, the available vaccines work effectively in people who are not yet infected, and not in those already infected. Secondly, there are challenges associated with reaching adequate levels of vaccine coverage. Experts estimate it will require vaccination rates of about 80 to 85% for the US population to be adequately protected against COVID-19 related hospitalizations and deaths [24–26]. It will take time to vaccinate enough people to reach herd immunity or to eradicate the disease, especially given the 2-dose regimen recommended for some of the current vaccines. Additionally, there has been inequitable global distribution of vaccines according to WHO, with high and upper middle-income countries receiving the lion’s share of the available doses in comparison to developing countries. More initiatives such as COVAX, created by WHO and earmarked for provision of vaccines to the developing world, are needed to continue to improve equitable access to vaccines in the future [27–30]. Another issue arises from individuals who will not be vaccinated even when given access, including immunocompromised patients who cannot take the vaccine, as well as those who choose to defer or delay vaccination due to vaccine hesitancy. Recent reports show that a significant fraction of Americans is unwilling to take the vaccine, and the recent pauses of Janssen and Astrazeneca vaccines could worsen this hesitancy and derail plans to reach herd immunity [24, 31–33]. These unvaccinated populations will inevitably become reservoirs and factories for ‘fitter’ virus species to evolve and emerge, potentially undermining the efforts to fight or eradicate the disease.

Perhaps of greatest concern is the emergence of new variants with reduced sensitivity to neutralization by vaccine-induced antibodies, as these variants represent the greatest threat to protective efficacy of current vaccines. As of the time of writing, there are 4 major variants of concern (VOC) in the world, namely Alpha (B.1.1.7), Beta (B.1.351), Gamma (P.1), Delta (B.1.617.2). Additional variants of interest are currently under surveillance including Lambda (C.37) [34–36]. Importantly, VOCs beta, gamma and delta harbor mutations in their Spike protein, such as E484K and L452R, that confer higher virulence, re-infection rates and resistance to sera from individuals vaccinated with Pfizer, Moderna and other major available vaccines [36–45]. Recently, reports by WHO and Eurosurveillance have demonstrated that the aforementioned VOCs have spread from their origins to hundreds of countries across the globe, with some instances showing that they can rapidly become the dominant source of new infections over the original SARS-CoV-2 variant [36, 46, 47]. The emergence of these new strains evokes fear of more deadly viral diseases breaking out in the future.

Therefore, there remains an urgent need for additional therapeutic strategies that work alongside these vaccines. Early translational efforts targeted against COVID-19 focused primarily on identifying treatments for the critically ill, as evidenced by several drug repurposing campaigns that tested antivirals and immune modulators in hospitalized patients. Most of the antivirals tested targeted the more advanced checkpoints of the virus life cycle, such as translation and RNA replication. Redemsivir is one agent that emerged from these studies, and is now used to treat hospitalized patients who require supplemental oxygen [48–50]. However, we have also learned that treating unhospitalized COVID-19 patients early improves prognosis. This highlights the importance of identifying more therapies for early management. Furthermore, some outpatients who eventually recover without medical intervention will experience long term-effects such as fatigue, loss of taste and smell, as well as cognitive impairment and cardiopulmonary dysfunction [19, 51]. These patients are not eligible for remdesivir treatment, and developing therapies for this population has the potential to improve recovery and quality of life. Antibodies and other viral entry inhibitors are suitable candidates for addressing these concerns, as they have the potential to minimize hospitalizations, prevent chronic late effects, and slow down spread by shortening the period of infectiousness and reducing mortality. While vaccines help to prevent new infections, entry inhibitors complement these gains by fighting COVID-19 in the unvaccinated population and the vaccinated who develop breakthrough infections. Moreover, the use of antibody cocktails and other viral entry inhibitors is also showing potential in combating new variants and other coronavirus clades. Below, we discuss the potential of inhibiting SARS-CoV-2 entry with antibodies and with alternative entry inhibitors such as recombinant human soluble ACE2, miniproteins, peptides and small molecules.

## Targeting Spike, ACE2 and/or their interactions

**Fig. 2:**
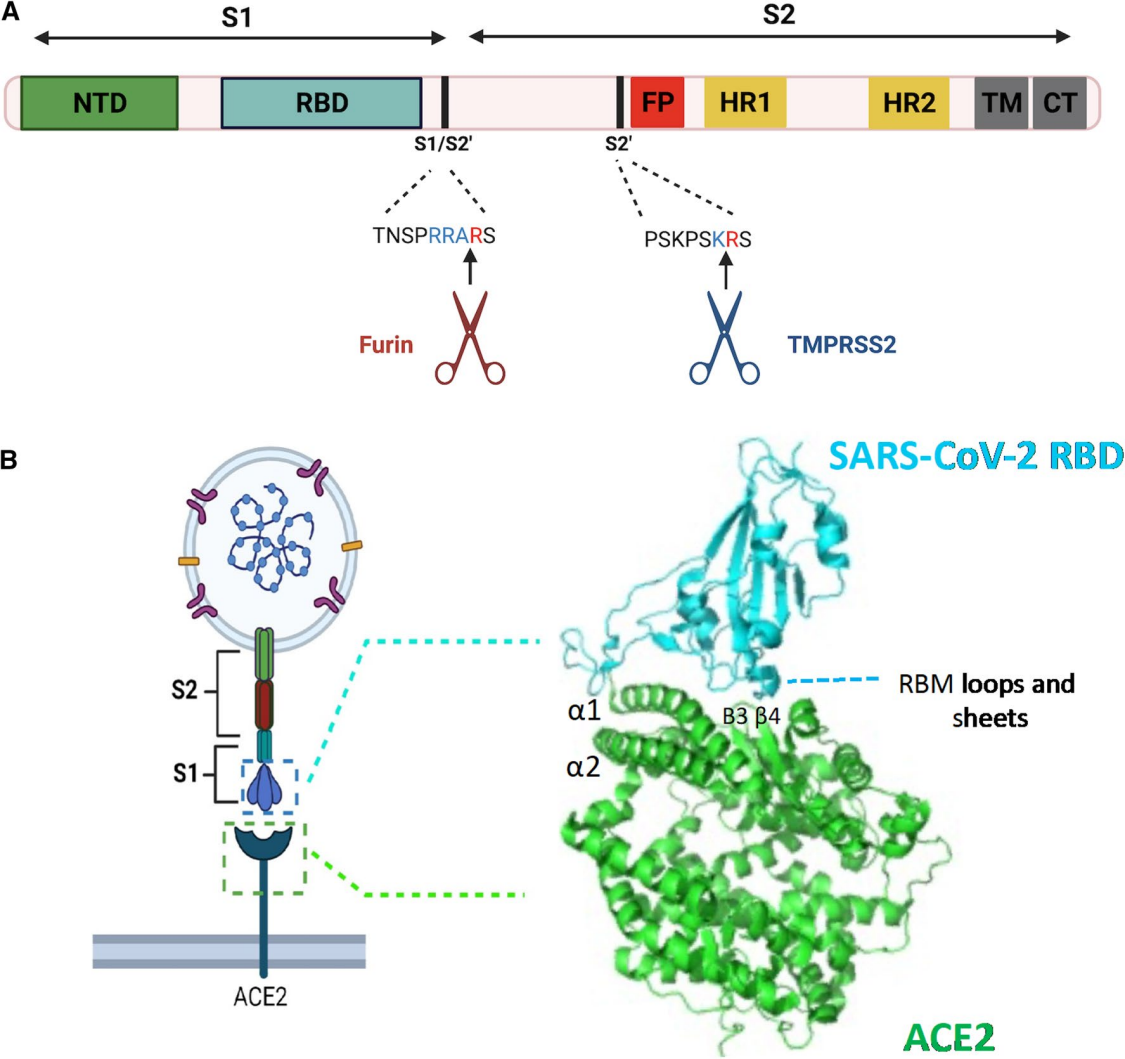
SARS-CoV-2 Spike glycoprotein domain structure and Spike interaction with ACE2. **a** Domain structure of the SARS-CoV-2 Spike comprises two subunits, S1 and S2. S1 consists of the NTD, RBD domains and the RBM within the RBD. Two cleavage sites, S1/S2’ and S2, are needed for priming and S activation for fusion of S2 and the cellular membrane to occur. S1/S2’ is cleaved by furin and S2’ by TMPRSS2 at the indicated cleavage sites. S2 subunit consists of FP; HR1 and HR2, TM, and the cytoplasmic tail (CT). **b** The interaction of Spike and the ACE2 receptor is defined by binding of S1 RBD and ectodomain motifs of ACE2. Specifically, the RBM of S1 RBD engage residues mainly from the α1 and α2 and β3/β4 binding motifs. (Figure created using Biorender)

SARS-CoV-2 Spike is a homotrimeric protein found on the surface of the viral membrane [52, 53]. Each monomer consists of two subunits, S1 and S2, located at the N- and C- termini, respectively. S1 consists of the N terminal domain (NTD) and receptor binding domain (RBD). Within the RBD is a sub-domain called the receptor binding motif (RBM) [54, 55]. The virus makes first contact with target cells that express ACE2 by way of a direct contact between the S1 RBD and ACE2 [56]. This receptor binding and virus attachment is the first step of viral entry into the cells, and therefore an attractive therapeutic target (Fig. 1). A subsequent step involves fusion with cell membrane to facilitate the release of the viral genome into the cell cytoplasm, and is mediated by the S2 subunit. S2 consists of the fusion peptide (FP), two α-helical heptad repeats (HR1 and HR2), a loop region, a transmembrane (TM) domain and cytoplasmic tail (CT). Binding of ACE2 to S1 induces a conformational change and processing that allows the FP to insert into the host cell membrane [54, 55]. The HR1 repeats, one from each subunit of the trimer, will refold together with the HR2 repeats in an anti-parallel fashion to form a coil-coil fusion core called the six-helix bundle (6HB). This structure brings the viral and cellular membranes together and drives cell fusion and release of the viral contents into the cytoplasm [54, 55]. This step is crucial and requires completion in order for the virus to achieve productive infection. Therefore, it represents another entry checkpoint that can be selectively targeted. The domain structure of the Spike glycoprotein and binding motifs of ACE2 are shown in Fig. 2. Various therapeutic proteins and their derivatives and small molecules have been employed to target these two distinct steps of SARS-CoV-2 entry as discussed in the following sections.

### Neutralizing antibodies

In August 2020, convalescent plasma (CP) was approved by the FDA for emergency use in COVID-19 patients. CP consists primarily of neutralizing antibodies from individuals who have recently recovered from SARS-CoV-2 infection. Therefore, these antibodies have the potential to help block entry of the virus into the cells and to facilitate viral clearance. CP has been used in several past outbreaks of other pathogens, and is generally understood to prevent infection and shorten duration and severity of the illness [4, 57, 58]. However, in the case of COVID-19, evidence of clinical benefit derived from CP has thus far been inconsistent, due to a lack of well controlled studies and the challenges associated with CP such as heterogeneity of plasma, lack of standardized protocols in preparing the antibody titers and how best to administer this plasma [59–61]. In contrast, synthetic antibodies, including monoclonal antibodies (Mab) can overcome some of these limitations as they are more specific, homogenous and scalable in terms of production. These types of antibodies can be generated from convalescent plasma, transgenic mice, B cell isolation or phage display libraries [15, 62, 63]. Since COVID-19 emerged, the field of Mabs has experienced an explosion of discoveries. Evidence shows that the majority of Mabs neutralize the virus by binding to epitopes in the RBD and preventing its interaction with ACE2. Characterization using techniques such as bio-layer interferometry (BLI) and surface plasmon resonance (SPR), as well as X-ray/cryo-EM structural studies reveal that this antagonism of ACE2 binding is enabled by the ability of antibodies to bind with high affinity and specificity to RBD [62–65]. These findings in turn have been supported by studies that show neutralization of infection of pseudotyped and live SARS-CoV-2 in vitro*,* as well as therapeutic protection of rodents and primates from virus-induced lung injury [15, 55–58]. Prominent examples of antibodies that have been characterized in this way include CCL12.1, 311mab-31B5 and 311mab-32D4, CR3022, S309, B38, CB6 and 4A8 [15, 62–64]. Some of these will progress to clinical trials soon, and several more are already being evaluated for therapeutic benefit in clinical trials including CT-P59, VIR-7831, AZD7442, TY027, SCTA01, and SAB-185 [15, 62–64, 66].

Currently, neutralizing monoclonal antibodies by Regeneron (casirivimab and imdevimab or REGEN-COV) and Eli Lilly (bamlanivimab and etesevimab) have already been granted emergency use authorization (EUA). Approval for REGEN-COV was obtained in November 2020, and the Eli Lilly combination was recently authorized in February 2021 [67, 68]. Clinically, these antibody regimens have demonstrated capacity to reduce viral load and hospital visits and are currently prescribed for treatment of mild to moderate COVID-19 in patients who are at risk for progressing to severe disease [67–69]. As their clinical efficacy continues to be monitored, the ongoing antigenic drift that poses ongoing challenges to vaccine efficacy also threatens to limit the efficacy of antibodies. A number of studies have reported findings that the new variants, particularly those that contain the E484K mutation such as the B.1.351 and P.1, display significant resistance to the efficacy of neutralizing Mabs [70–72]. This is particularly true when the antibodies are used as monotherapies [72–74]. Indeed, the US government has now warned against use of bamlanivimab alone, which was initially approved as a monotherapy, and now recommends bamlanivimab use together with etesevimab [75]. The individual antibodies in the two EUA cocktails recognize distinct epitopes and their combinatorial use limits the development of escape mutants and resistance. New data has shown that the bamlanivimab and etesevimab combination has relatively higher neutralization efficacy against variants compared to either antibody alone, whilst REGEN-COV has largely maintained its potency against all the variants tested so far [69, 76, 77]. These observations validate the use of cocktails and emphasize the importance of designing antibodies from more conserved epitopes to counter neutralization escape mutations as well as the need to create broad-spectrum antibodies and other therapies for future variants and outbreaks.

Fortunately, the development of biologics with a wide neutralization breadth is already a growing area of research. Rappazzo et al. have shown that antibodies engineered using directed evolution can be broadly active. Specifically, one of their affinity matured variants, ADG-2, which recognizes a highly conserved epitope exhibited potent neutralization against authentic SARS-CoV-2 in vitro*,* and protected mice infected with SARS-CoV and SARS-CoV-2 against viral replication and lung pathology. More importantly, when compared to EUA antibodies that neutralized mostly SARS-CoV-2, ADG-2 displayed a wider breadth against clade 1 sarbecoviruses including SARS-CoV, SARS-CoV-2, WIVI, LYRa11, Rs4231, GD-Pangolin and Pangolin-GX-P2V [78]. Another study by Wec et al. has also identified several antibodies from a convalescent Covid-19 patient that cross-neutralized SARS-CoV, SARS-CoV-2 and WIVI [79]. More recently, two studies have reported similar discoveries. Starr et al. discovered antibodies that target conserved, functionally constrained RBD residues. One of these, S2H97, showed high affinity and neutralization breadth across SARS-CoV-2-related sarbecoviruses [80]. An accompanying study showed that S2X259, which binds to a highly conserved cryptic RBD epitope, cross-neutralized all the VOCs and a wide spectrum of human and zoonotic sarbecoviruses. Notably, prophylactic dosing of Syrian hamsters with S2X259 offered protection against a SARS-CoV-2 and B.1.351 variant challenge [81]. Additional antibodies that have demonstrated similar efficacy against variants are summarized in Table 1 [82–84].

**Table 1:** Prominent examples of viral entry inhibitors that have demonstrated therapeutic or prophylactic efficacy in cross-neutralization, suppression of escape mutants and broad activity against circulating variants and sarbecoviruses

Inhibitor type	Agent	Study design/model	Main findings	References
Antibody	ADG-2	Pseudotypes and WT SARS viruses in vitro and in vivo	Neutralized SARS-CoV, SARS-CoV-2, bat SARSr CoVs, sarbecoviruses and protected mice against SARS-CoV and SARS-CoV-2	[78]
	ADI-55689	Pseudotypes and WT SARS viruses in vitro	Neutralized SARS-CoV, SARS-CoV-2, bat SARS-like WIVI in cells	[79]
	S2H97, S2X259	Pseudotypes and WT SARS viruses in vitro and in vivo	Neutralized SARS-CoV-2, all sarbecovirus clades. Prevented escape mutants and neutralized all VOC. Protected Syrian hamsters from SARS-CoV-2 and B.1.351	[80, 81]
	REGN10987 + REGN10933	SARS-CoV-2 pseudotypes in vitro	Agents prevented selection of escape mutants in vitro	[82]
	S309 + S304	Pseudotypes and aunthentic SARS viruses in vitro	Neutralized SARS-CoV-2, SARS-CoV, WIVI pseudotypes as well as live SARS-CoV-2 in cells	[83]
	CV38-142 + COVA1-16	Pseudotypes and aunthentic SARS viruses in vitro	Neutralized SARS-CoV, SARS-CoV-2, B.1.1.7 and B.1.351 in cells	[84]
Nanobody	Multiple candidates (e.g. VHH VE)	Pseudotypes and WT SARS viruses in vitro	VE neutralized SARS-CoV, SARS-CoV-2 and escape mutants	[89]
	Multiple candidates (NB34, 36,N105)	Pseudotypes and WT SARS viruses in vitro	Neutralized SARS-CoV-2 and variants including B.1.1.7 and B.1.351 in cells	[90, 94]
	Multiple candidates (Nb30, Nb56 trimers)	Pseudotypes in vitro	Neutralized SARS-CoV-2 and VOC (UK and South African variants) in cells	[91]
	Multiple candidates (S1-1, S1-RBD-15)	Pseudotypes and WT SARS viruses in vitro	Neutralized SARS-CoV, SARS-CoV-2, B.1.351 and escape mutants in cells	[95]
	Multiple candidates (e.g. WNb 2 + 7)	Pseudotypes and WT SARS viruses in vitro and in vivo	Neutralized SARS-CoV-2 in vitro, N501Y D614G variant in vitro and prophylactically reduced viral loads in mice	[96]
Decoy receptor	sACE22.v2.4	Pseudotypes and WT SARS viruses in vitro	Neutralized various ACE2-utilizing SARS-related viruses from humans and bats in cells	[102]
	CTC-445.2d, CTC-445.2t	Pseudotypes and WT SARS viruses in vitro and in vivo	Showed resilience to escape mutants; neutralized SARS-CoV-2 in vitro. Protected mice and hamsters against SARS-CoV-2	[103]
	ACE2(740)-Fc	Pseudotypes and WT SARS viruses in vitro	Neutralized SARS-CoV-2 and other ACE2-utilizing CoVs in cells	[102]
	LCB1	WT SARS viruses in vitro and in vivo	Neutralized WT SARS-CoV-2 in vitro and prophylactically protected mice against SARS-CoV-2, B.1.1.7 and E484K/N501Y variant	[106, 107]
Fusion inhibitor	EK1, EK1C4	Pseudotypes and WT SARS viruses in vitro and in vivo	Inhibited entry of various CoVs including SARS-CoV-2, SARS-CoV, MERS, WIVI, HCoV-NL63, HCoV-0C43 in vitro. Protected mice from MERS, HCoV-0C43, SARS-CoV-2	[122, 123]
	IPB-01, IPB-02	Pseudotypes of SARS viruses in vitro	Inhibited SARS-CoV and SARS-CoV-2 entry in cells	[124]
	(SARS_HRC_-PEG_4_)_2-chol_	WT SARS-CoV-2 in vitro and in vivo	Inhibited SARS-CoV-2 entry in vitro and prophylactically protected ferrets from SARS-CoV-2 infection	[125]

However, it is not only antibodies that are demonstrating success in dealing with current or potential escape mutants. Nanobodies are also proving to be a viable option. Nanobodies are single domain antibodies that are generated from immunized llamas, camels and phage displays [85–88]. Recent published evidence shows that multivalent nanobodies are capable of both neutralizing circulating variants and preventing emergence of resistant escape mutants via binding to multiple, non-overlapping epitopes, avidity effects and binding to conserved epitopes largely inaccessible to normal antibodies [89–96]. Table 1 summarizes the main findings from these studies. Additionally, nanobodies have properties that may be beneficial considering the potential use of monoclonal antibodies as pre-and post-exposure prophylactics (PEPrs). Pre-clinically, monoclonal antibodies have prophylactic value in addition to therapeutic value. Widespread evidence of prophylactic protection against SARS-CoV-2-related respiratory injury in animal models ranging from mice to hamsters to rhesus macaques has been reported [97–101]. Consistently, preliminary evidence from ongoing clinical trials with EUA monoclonal antibody therapies is also very promising [102–104]. However, it is important to point out that widespread outpatient use of potential PEPrs therapies would be most practical with agents that can be conveniently administered. Antibodies are molecularly large, less stable, complex and costly to produce. Currently, antibodies are usually given intravenously in healthcare facilities that must also be equipped with resources for dealing with potential infusion reactions. Nanobodies, on the other hand are smaller, cheaper to make and can be nebulized for easier and more convenient pulmonary delivery using inhalers or nasal sprays [63, 85]. Collectively, these facts make monoclonal antibody cocktails, broad-spectrum antibodies and multivalent nanobodies the future in terms of dealing with variants during early onset of disease and prevention of infection pre- and post-exposure.

### Recombinant human soluble ACE2 and other protein-based antivirals

**Fig. 3:**
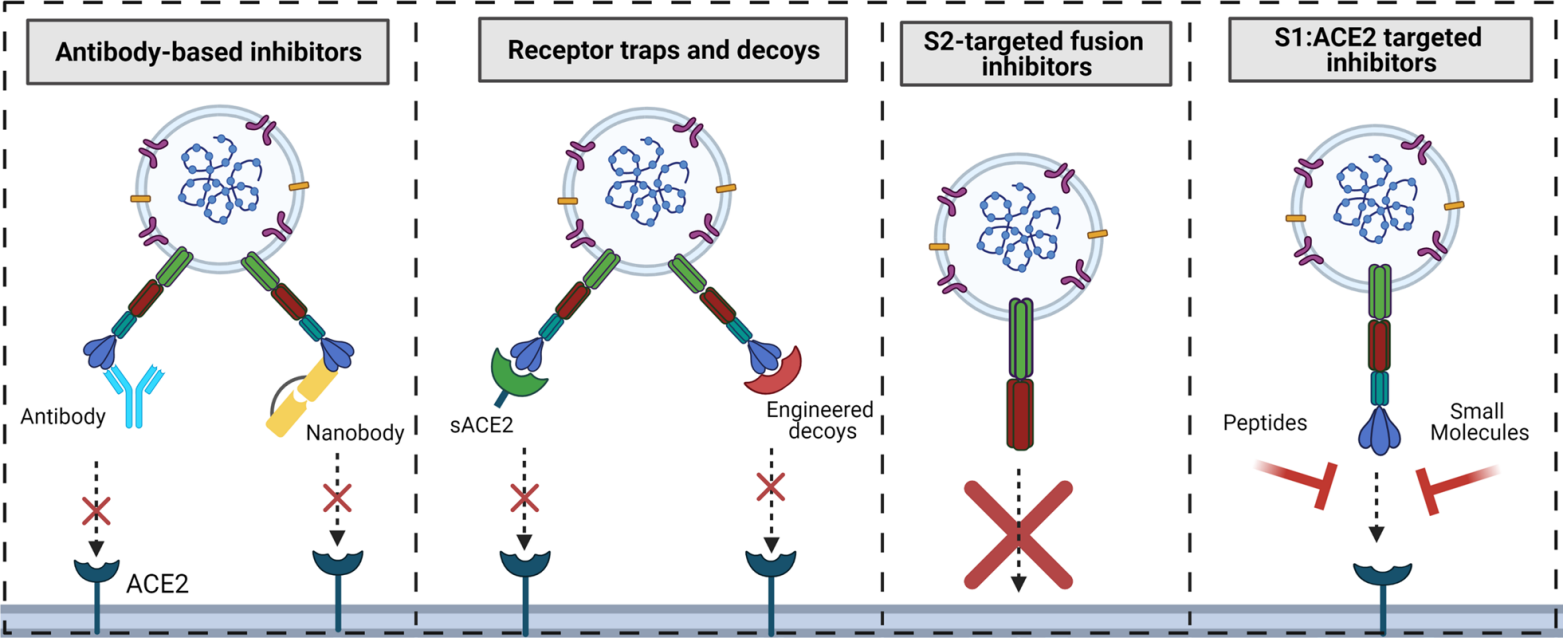
Summary of strategies of targeting viral entry at the surface membrane. Four approaches are highlighted including antibody-based inhibitors that consists of monoclonal antibodies and nanobodies. Receptor decoys consists of WT soluble ACE2 or versions of ACE2 that are engineered to have high affinity than WT ACE2. Various inhibitors mainly based on HR2 of S2 have also been designed to prevent fusion of the S2 with cellular membrane. Also, peptides and small molecules that are designed to interfere with the S1 RBD and ACE2 interaction have also been made. (Figure created using Biorender)

The use of protein-based antivirals has been dominated by antibodies or their functional fragments that bind to the RBD of S1. An alternative to this strategy is to target the ectodomain of ACE2, as it serves as the SARS-CoV-2 receptor. These decoy receptors (Fig. 3) work like scavengers that have the potential to outcompete the endogenous transmembrane ACE2 for binding to Spike [15]. Moreover, although escape mutants can sometimes outmaneuver antibody defenses with RBD- or NTD-specific mutations, it is more difficult to escape decoys without also losing virulence, since decoy receptors have the same binding interface as does the endogenous ACE2. Furthermore, soluble ACE2 has already been found to be safe as shown in clinical studies focused on treatment of ARDS and SARS [15]. It is expected, therefore, that soluble ACE2 receptors will likely be safe and potentially effective against SARS-CoV-2 infection. An earlier pre-clinical study by Monteil et al. using clinical grade soluble recombinant human ACE2 (hrACE2) confirmed this potential, and showed that hrACE2 prevented infection by SARS-CoV-2 significantly [105]. A number of ongoing clinical trials are currently evaluating the potential of soluble rACE2 [15]. For example, rhACE2 APN01 is now in Phase II clinical trials. Phase I data showed that APN01 can reduce viremia and viral titers, and preliminary evidence from phase II data indicates that APN01 lowers risk of medical complications and shortens recovery time [106, 107]. These exciting findings have inspired other groups to engineer even more potent forms of soluble ACE2 using computational design, deep mutagenesis and affinity maturation. A study by Chan et al. shows that soluble ACE2 designed using affinity maturation based on the mutations of the 117 residues involved in the binding of S led to the discovery of sACE22.v2.4. sACE22.v2.4 was more potent than WT sACE2, and its resilience against mutants was exemplified by ability to potently neutralize coronaviruses that use ACE2 as entry port including SARS-CoV-2, SARS-CoV and SARS-like bat coronaviruses [108]. Two other studies by Glasgow and Linsky et al. have employed a similar approach with success [109, 110]. In particular, two decoys engineered by Linsky et al., namely CTC-445.2d and CTC-445.2t, showed potent neutralization of SARS viruses and protected Syrian hamsters from SARS-CoV-2 following a single prophylactic dose [110]. More importantly, other findings have shown that even smaller versions of decoy receptors can yield potent neutralization effects [111]. Hyper-stable miniprotein binders that include AHB1, AHB2, LCB1 and LCB3 have displayed impressive in vitro inhibition of SARS-CoV-2 infection with potencies in the nano- to picomolar range [112]. LCB1, only 56 residues, has been utilized as the lead binder in follow up studies to evaluate in vivo efficacy when administered either intraperitoneally (LCB1-F_c_) or intranasally (LCB1v1.3) in a transgenic COVID-19 mouse model. LCB1 administration using both routes protected the mice post-exposure against SARS-CoV-2-mediated lung disease as well as pre-exposure, even when dosed intranasally as many as five days before virus inoculation. Notably, LCB1v1.3 protected the mice in vivo against the B.1.1.7 variant and a variant encoding key E484K and N501Y mutations in Spike following prophylactic dosing through the nose [113]. Taken together, these protein-based antivirals hold clinical promise and point to a remarkable therapeutic and prophylactic potential now, as well as potential protection against re-emerging ACE2-utilizing coronaviruses in the future.

### S1 and S2 targeted peptides

Peptides represent another type of inhibitor that can be directed against Spike and ACE2 to prevent viral entry. Peptides are smaller, simpler and cheaper to make than are antibodies or the other protein-based antivirals. Their well-known liability is generally low bioavailability due to degradation and metabolism when given systemically [113]. However, as a COVID-19 therapeutic, this disadvantage can easily be overcome through nebulization or dry aerosol powders for direct delivery to the lungs [113]. In general, we can divide SARS-CoV-2 Spike-targeted peptide inhibitors into two groups: those that perturb S1 RBD: ACE2 binding, and those that interfere with fusion of S2 with the membrane (Fig. 3). Previous studies by groups such as the Huang and Cho labs had shown that peptides extracted from important S1 RBD-recognizing motifs in ACE2 (*see* Fig. 2b), such as those in the N-terminal helix (α-1), can result in significant competitive antagonism and antiviral activity [114, 115]. For example, the Cho group showed that linking together two non-contiguous segments that are close in space can inhibit SARS-CoV infection with a half-maximal inhibition concentration of 100 nM [114]. Other studies also reported similar findings with S1-derived linear peptides [116, 117]. Given the similarity in the binding conformation between S1 RBD of SARS-CoV and SARS-CoV-2 with ACE2 and the high sequence identity of the S1 RBD of SARS-CoV and SARS-CoV-2, there is reason to believe that peptides against ACE2: S1 RBD binding in SARS-CoV-2 can also be effective [62]. Findings by Karoyan et al. appear to corroborate this expectation. Their data show that peptide fragments (P8, P9, P10) from the α-helix of the ACE2 peptidase domain (PD) that are rationally modified with residues that have a propensity for helical folding show high binding affinity and antiviral activity against authentic SARS-CoV-2 in the nanomolar range [118]. A study by Curreli et al. also showed that peptides from a similar region of ACE2 that are structurally stabilized with double stapling show inhibitory activity against pseudotyped and live SARS-CoV-2 in the low micromolar range [119]. For peptides that are based on the binding motif of S1 RBD, particularly the RBM as shown in Fig. 2b, one lab has reported a group of peptides called SARS-BLOCK™ with sub-micromolar antiviral activity against SARS-CoV-2 pseudovirions [120]. On the other hand, some studies report more modest activity or complete lack of activity of peptide inhibitors. For example, the Zhang lab published that even though a 23-mer peptide from the α-helix of PD of ACE2 exhibited high binding affinity in the nanomolar range, it lacked appreciable competitive capability against soluble ACE2 for binding S1 RBD [121, 122]. Certainly, an argument can be made that the lack of binding here may be due to limited secondary structure in solution of the linear native peptide designed by Zhang et al.[113]. Nonetheless, a different group has shown that even with stapling that dramatically improved helicity of their peptides, no appreciable binding activity was observed for either stabilized and non-stabilized peptides [123]. In our lab we have found that peptides rationally designed from the binding motifs of either ACE2 or S1 RBD display modest inhibitory activity in the low micromolar range (unpublished). These inconsistencies therefore warrant more data for safer conclusions to be reached regarding the activity of peptides that inhibit S1 RBD: ACE2 interaction and their prospects as COVID-19 therapeutics.

As noted above, viral fusion with the cellular membrane also represents a point of potential therapeutic targeting. Since both HR1 and HR2 are needed to come together to form the 6HB and then to fuse, designing a peptide mimicking one region will competitively interfere with formation of the fusion core [54, 55]. This approach has been utilized to prevent entry of other viruses with heptad regions such as HIV. In fact, enfuvirtide is a fusion inhibitor that is approved for treating HIV infection [113]. HR2 is usually used as template to make HR1-directed peptides, and this approach has been successfully applied for coronaviruses [113]. Much of this work was published before the inception of SARS-CoV-2, and targeted viruses such as SARS-CoV, MERS-CoV and HCoV-229E [124, 125]. Perhaps the most impressive results were obtained from OC43-HR2P, as reported in 2019 [126]. OC43-HR2P peptide was derived from the HR2 domain of HCoV-OC43, and showed broad spectrum activity against alpha- and beta-coronaviruses. An optimized version of OC43-HR2P from this study (EK1) was quickly tested once SARS-CoV-2 emerged, and showed potent activity against SARS-CoV-2 infection in vitro. A lipid-conjugated form of EK1 called EK1C4 with an IC_50_ of 37 nM against SARS-CoV-2 infection in vitro has also been tested in mice. In the mouse study, EK1C4 displayed not only a good in vivo safety profile, but also antiviral activity and metabolic stability following intranasal administration [127, 128]. The extension of activity from previous hCoV strains such as SARS-CoV stems from the high conservation of the HR regions. For instance, HR1 and HR2 of SARS-CoV and SARS-CoV-2 have 92.6% and 100% similarity, respectively [113]. The conservation allows for broad spectrum activity against hCoVs. Other HR2-derived peptides have also been identified and tested against SARS-CoV. IPB-01 and IPB-02 have shown low nanomolar activity against infection with SARS-CoV and SARS-CoV-2 pseudovirions [129]. Another lipid-modified fusion peptide called (SARS_HRC_-PEG_4_)_2-chol_ inhibited SARS-CoV-2 with a half maximal inhibitory concentration of 3.8 nM, and intranasal administration protected ferrets from SARS-CoV-2 infection [130]. The pan-specific activity and in vivo protection of animals show that fusion inhibitors have potential for clinical utility. Altogether, peptides are a promising therapeutic option for COVID-19 in the future, though more research is needed.

### Small molecules inhibiting the ACE2: S1 RBD interaction

Small molecules therapeutics generally are better situated to overcome problems such as cell permeability and metabolic lability than are peptides, but their development also takes time. Thus far, efforts to develop small molecule therapeutics for COVID-19 have largely involved repurposing antiviral drugs already approved for clinical use, or which have undergone regulatory processes tied to clinical trials. The drug remdesivir, previously clinically studied for Ebola, was identified in this manner. Additional antiviral drugs for RNA viruses targeting the RdRP, helicase and protease proteins are undergoing further clinical evaluation for efficacy against COVID-19 [48, 131]. The same approach can be adopted for viral entry inhibitors. Unfortunately, the literature shows that most small molecule inhibitors that were previously evaluated as entry antagonists have no regulatory approval. In addition, the reported pre-clinical potency is largely in the low micromolar range, implying that most of these candidates will first have to be tested in the context of SARS-CoV-2 and then be optimized for affinity and potency [132–134]. Examples of inhibitors that target S1 RBD and ACE2 and their interactions in the context of ACE2-utilizing coronaviruses include cepharantine, VE607, SSAA09E2, emodin, HTCC and HM-HTCC [132–134]. Drug reprofiling studies in our lab that evaluated candidates targeting the ACE2: S1 RBD interaction showed that of those tested, cepharantine was the most promising candidate, with single digit micromolar potency against SARS-CoV-2 RBD binding to ACE2 (unpublished). Indeed, several findings in recent publications have validated these observations and demonstrated that cepharantine does display anti-viral activity against both pseudotyped and authentic SARS-CoV-2 infection in vitro, with potencies ranging from 0.73 µM to 30 μM [135–141]. Additionally, some candidate small molecule inhibitors with novel activity against coronaviruses have also been identified. Hanson et al. discovered coriligan through a high content screen that inhibited the RBD and ACE2 interaction with an IC_50_ of 5.5 µM [142]. In a study by Day et al., an SPR based RBD: ACE2 screen was done on 3,141 compounds. In vitro studies using live SARS-CoV-2 showed that the hit compounds suramin and evans blue possessed antiviral activity with acceptable selectivity and IC_50_ values of 46 and 28 μM, respectively [143]. Overall, compared to the other studies discussed above, targeting ACE2 and S1 RBD interaction with small molecules remains a developing area of research. The reported antiviral potencies thus far are modest, indicating the need for significant additional optimization to support their development into efficacious agents. The strategy of using small molecules and other agents to prevent viral entry through the cell surface membrane is summarized in Fig. 3.

## Host proteases and endosome acidification inhibitors

Although S1 and S2 mediate viral attachment and membrane fusion to enable the virus to unload its genetic cargo, function of these two subunits is enabled by the participation of at least 3 types of host proteases: furins, cathepsins and surface serine proteases. Viral entry generally occurs either through direct fusion of the virus with the surface membrane or endocytic uptake, and what determines which proteases will dominate in facilitating fusogenic activity is the entry pathway utilized [54, 55, 108, 144]. The SARS-CoV-2 Spike protein has two cleavage sites, S1/S2’ and S2’. For non-endocytic entry, the S1/S2’ site is cleaved primarily by the furin proprotein convertase. This cleavage may then help to reveal the S2’ site more fully to the surface trypsin-like serine proteases such as TMPRSS2. The S2’ site is immediately upstream of the fusion peptide (FP), and its cleavage by TMPRSS2 exposes the hydrophobic peptide (FP) for insertion into the membrane (Fig. 2a) and subsequent formation of the 6HB as already described. Conversely, if the virus takes the endocytic route, cathepsins will play a more dominant role [54, 55, 108, 144]. Specifically, the cathepsin L isoform has been shown to be more important in S2’ cleavage for coronaviruses. Cathepsin L is a lysosomal cysteine protease and its function, like that of many other cathepsins, is pH-dependent, with optimal pH activity ranging from 3–6.5 [114, 144, 145]. Without cleavage of Spike by these proteases, the virus would not be able to fuse with the lysosomal or autolysosomal membrane to release its genome into the cytoplasm (Fig. 1). Therefore, all the three different classes of proteases noted above represent rational targets for COVID-19 therapeutic intervention.

### Furin and TMPRSS2 inhibition

Furin inhibitors have previously been reported as possible targets in the context of other viruses such as influenza, and may also be relevant for SARS-CoV-2. SARS-CoV Spike contains only the monobasic S2’ site, and not the extra polybasic (RRAR) motif for the S1/S2’ site found in SARS-CoV-2 (Fig. 2a). The S1/S2’ in SARS-CoV-2 likely plays an activating role, which might contribute to the higher pathogenicity and multi-organ infectivity of SARS-CoV-2 [11, 144]. A common way to inhibit S1/S2’ site processing is to design peptide substrate mimics. The consensus sequence recognized by furin proteases is R-X-R/K-R_↓_ and studies in the past have shown that the peptidomimetic decanoyl-RVKR-chloromethylketone (dec-RVKR-cmk) inhibits furins and cleavage of viral glycoproteins [144, 146]. In SARS-CoV-2 studies, dec-RVKR-cmk inhibited infection in vitro with an IC_50_ of 5 μM [147]. MI-1851, another furin inhibitor has also been found to reduce SARS-CoV-2 titers in Calu-3 cells by almost 200-fold at 10 µM [146]. For TMPRSS2, various inhibitors, both peptidomimetics and small molecules, have been reported for previous coronavirus strains such as MERS and SARS-CoV [144, 147, 148].The peptidomimetic inhibitors that have shown promising activity against SARS-CoV-2 include aprotinin, MI-1900 and MI-432. Aprotinin has been tested previously in the clinic for combating influenza infection, and has also shown significant inhibition of SARS-CoV-2 growth at 10 μM [144, 149]. MI-1900 and MI-432 have both shown higher potency compared to aprotinin under similar experimental conditions and are thus more promising. More importantly, the combination of MI-1851 plus MI-432 was viable and more effective than either therapy alone [146]. Equally promising are the small molecule inhibitors of TMPRSS2, camostat and nafamostat mesylate. Camostat and nafamostat mesylate are analogues with clinical approval for pancreatitis and disseminated intravascular coagulation [150]. Indeed, camostat was one of the early small molecule inhibitors to be shown to have significant activity in blocking the entry of SARS-CoV-2 into cells [56]. However, nafamostat is actually the more potent analogue, and has been shown to inhibit SARS-CoV-2 replication in Calu-3 cells with an EC_50_ of 10 nM [145, 147]. Both inhibitors are currently in clinical trials for evaluation as COVID-19 therapeutics, and results regarding their efficacy are eagerly awaited [56, 151].

### Cathepsin inhibition

A number of cathepsin inhibitors against coronaviruses have also been reported in various studies. Amongst them are teicoplanin, K1777, SSAA09E1, SID-26681509 and P9 derivatives [64, 113, 144, 152, 153]. Teicoplanin has exhibited good activity against SARS-CoV-2 pseudovirions entry with an IC_50_ of 1.6 µM [154]. The same can be said for SID-26681509 and P9 derivatives. A study by Ou et al. found that the Cathepsin L inhibitor, SID 26681509, independently decreased SARS-CoV-2 S pseudovirion entry by about 76% at 2 μM [155]. The P9 derivates, P9R and 8P9R, have also shown significant activity against SARS-CoV and SARS-CoV-2 ranging in the low micro- to nanomolar range [156, 157]. More importantly, 8P9R demonstrated antiviral activity by decreasing the SARS-CoV-2 viral load in vivo in mice and hamsters [157]. The inhibitors mentioned above, such as SID-26681509, inhibit the protease activity of cathepsins in a direct way by interacting with the enzyme active site through mimicking of the endogenous substrate. However, indirect inhibition of protease activity through pH modulation is also an option. Endosome acidification inhibitors act through this mechanism, and a number were highly touted as potential effective treatments at the beginning of the pandemic [152]. Such inhibitors, which include chloroquine, hydroxychloroquine and azithromycin, function by elevating the pH of the endosome, shifting the pH outside the optimal range and thereby indirectly suppressing cathepsin protease activity [158–160]. Despite this rational and promising pre-clinical activity, these inhibitors have not demonstrated evidence of consistent and robust benefit when evaluated in various clinical trials [158–163]. Given some of the known side effects of chloroquine derivatives, such as cardiac-related toxicities and retinopathy, their consideration for clinical use has now been put on hold [164]. Despite these recommendations against endosome acidification inhibitors, the other protease inhibitors remain potential candidates for clinical development given their specificity. Future studies will reveal and determine their utility as future COVID-19 therapeutics.

## Conclusions and future perspectives

COVID-19 is now understood as a biphasic illness, with an early viral phase and a more dangerous host-immune response phase. This knowledge has shaped our translational and clinical therapeutic strategies to find treatments for those infected. The ongoing antigenic drift of SARS-CoV-2 is also shaping the fight against COVID-19. Four major variant strains have now been identified, which have generally shown increased transmissibility and resistance to the efficacy of vaccines and monoclonal antibodies [36, 37]. Vaccines, particularly those that are mRNA-based, have shown that they offer some protection against the variants, albeit with reduced effectiveness, and multiple doses of the vaccines, including booster shots, may be necessary in the future [165–168]. Health officials will also continue to monitor variants of interest that have already been identified. In addition, the emergence of SARS-CoV-2 has also renewed fears that another zoonotic spillover will occur and cause an even more deadly outbreak. These fears are not unfounded, given that we experienced more than 10 serious outbreaks from emerging RNA viruses in the last 20 years alone [169]. Each of these aspects have subjected the counter-measures currently in place to increased attention, asking how such measures can be made more effective based on available evidence. In addition, and also of critical importance, is the development of future plans for dealing with mutant strains and potential outbreaks. In this review, we have highlighted the utility of vaccines and the gaps they leave in fighting COVID-19. We then demonstrated that the mechanism of action of entry inhibitors makes them suitable agents for early management of COVID-19 to help cover some of the gaps, and shown why continued research on such inhibitors is crucial. The monoclonal antibody entities are farthest along the drug development pipeline, with some already approved for use (EUA) and several more in advanced stages of clinical trials [66]. Cocktails of monoclonal antibodies, multivalent nanobodies and recombinant soluble ACE2 have also demonstrated therapeutic effect against mutant strains, including those currently in circulation, as well as broad, cross-family coronavirus efficacy. The antibody species’ ability to limit resistance and deliver broad activity comes from their targeting of more than one epitope, including some from the more conserved regions of Spike. For agents based on recombinant ACE2, similar efficacy comes from their similarity with the endogenous receptor, which makes it difficult for mutant strains to arise without also losing infectivity. In addition to these valuable therapeutic effects and their potential as agents to treat future outbreaks, these protein-based antivirals have also demonstrated they can be useful when given prophylactically, even several days before exposure. As noted earlier, various subgroups of people will benefit from prophylactic treatment using these agents. As for the miniprotein, peptide and small molecule therapeutics, current literature suggests that they are not as advanced in terms of clinical development as are the antibodies or recombinant ACE2. However, their utility is in their size and ability to be more readily developed into therapeutic formulations that can be self-administered either as oral pills or inhalants. More research is therefore still needed, as researchers and decision makers continue to evaluate the potential use of entry inhibitors for outpatient prophylaxis. Also, given that the nasal passage is the most dominant and initial site of infection, aerosolization can potentially be beneficial in preventing viral spread to the lungs through use of nasal sprays [8]. Finally, more investment in the development of entry inhibitor therapeutics as well as other antivirals and therapies directed against the host immune response is needed, as their availability will impact our options in responding not only to future SARS-CoV-2 lineages, but also to future coronavirus pandemics. Recent events have made it abundantly clear that it is both more impactful and cost effective to prevent or prepare for a pandemic like the one caused by COVID-19, than to encounter such a pandemic without preparation. For this reason, current proposals by the US and international community to invest more into pro-active and pre-emptive countermeasures against future outbreaks are commendable, as this development will shorten the time between an outbreak and an effective therapeutic response [170, 171].

## Data Availability

All materials available in this article.

## Abbreviations

6-HB: 6 Helix bundle
ACE2: Angiotensin converting enzyme 2
ARDS: Acute respiratory distress syndrome
BLI: Bio-layer interferometry
CDC: Center for disease control
COVID-19: Coronavirus disease 2019
CP: Convalescent plasma
CQ: Chloroquine
Cryo-EM: Cryogenic electron microscopy
CT: Cytoplasmic tail
EUA: Emergency use authorization
FDA: Food and drug administration
FP: Fusion peptide
HCQ: Hydroxychloroquine
HR1: Helical heptad repeat 1
HR2: Helical heptad repeat 2
hCoV: Human coronavirus
HCoV-229E: Human coronavirus 229E
HCoV-OC43: Human coronavirus oc43
HIV: Human immune deficiency syndrome
hrACE2: Soluble recombinant human ACE2
IFN-γ: Interferon gamma
Il-1β: Interleukin 1 beta (IL-1β)
IL-6: Interleukin 6
IL-8: Interleukin 8
Mab: Monoclonal antibody
MERS-CoV: Middle east respiratory syndrome coronavirus
NTD: N terminal domain
PD: Peptidase domain
PEPrs: Pre-and post-exposure prophylactics
RBD: Receptor binding domain
RBM: Receptor binding motif
RNA: Ribonucleic acid
RdRp: RNA dependent RNA polymerase
S1: Subunit 1
S2: Subunit 2
SARS: Severe acute respiratory syndrome
SARS-CoV-2: Severe acute respiratory syndrome coronavirus 2
SPR: Surface plasmon resonance
TMPRSS2: Transmembrane protease serine 2
TNF-α: Tumor necrosis factor alpha
TM: Transmembrane
US: United States
VOC: Variants of concern
WT: Wild type

## References

[1] Nicola M, Alsafi Z, Sohrabi C, Kerwan A, Al-Jabir A, Iosifidis C, Agha M, Agha R (2020). The socio-economic implications of the coronavirus pandemic (COVID-19): a review. Int J Surg.

[2] Cucinotta D, Vanelli M (2020). WHO declares COVID-19 a pandemic. Acta Biomed.

[3] Wiersinga WJ, Rhodes A, Cheng AC, Peacock SJ, Prescott HC (2020). Pathophysiology, transmission, diagnosis, and treatment of coronavirus disease 2019 (COVID-19): a review. JAMA.

[4] Izda V, Jeffries MA, Sawalha AH (2021). COVID-19: A review of therapeutic strategies and vaccine candidates. Clin Immunol..

[5] Sun P, Lu X, Xu C, Sun W, Pan B (2020). Understanding of COVID-19 based on current evidence. J Med Virol.

[6] Hu B, Guo H, Zhou P, Shi ZL (2021). Characteristics of SARS-CoV-2 and COVID-19. Nat Rev Microbiol.

[7] Harrison AG, Lin T, Wang P (2020). Mechanisms of SARS-CoV-2 transmission and pathogenesis. Trends Immunol.

[8] Hou YJ, Okuda K, Edwards CE, Martinez DR, Asakura T, Dinnon KH, Kato T, Lee RE, Yount BL, Mascenik TM (2020). SARS-CoV-2 reverse genetics reveals a variable infection gradient in the respiratory tract. Cell..

[9] Mason RJ. Pathogenesis of COVID-19 from a cell biology perspective. Eur Respir J. 2020, 55(4). 10.1183/13993003.00607-2020 PMC7144260 32269085

[10] Sungnak W, Huang N, Becavin C, Berg M, Queen R, Litvinukova M, Talavera-Lopez C, Maatz H, Reichart D, Sampaziotis F (2020). SARS-CoV-2 entry factors are highly expressed in nasal epithelial cells together with innate immune genes. Nat Med.

[11] Stratton CW, Tang YW, Lu H (2021). Pathogenesis-directed therapy of 2019 novel coronavirus disease. J Med Virol.

[12] Sette A, Crotty S (2021). Adaptive immunity to SARS-CoV-2 and COVID-19. Cell.

[13] Le Bert N, Clapham HE, Tan AT, Chia WN, Tham CYL, Lim JM, Kunasegaran K, Tan LWL, Dutertre CA, Shankar N *et al*. Highly functional virus-specific cellular immune response in asymptomatic SARS-CoV-2 infection. J Exp Med. 2021, 218(5). 10.1084/jem.20202617 PMC7927662 33646265

[14] Wang J, Jiang M, Chen X, Montaner LJ (2020). Cytokine storm and leukocyte changes in mild versus severe SARS-CoV-2 infection: review of 3939 COVID-19 patients in China and emerging pathogenesis and therapy concepts. J Leukoc Biol.

[15] Twomey JD, Luo S, Dean AQ, Bozza WP, Nalli A, Zhang B (2020). COVID-19 update: the race to therapeutic development. Drug Resist Updat..

[16] Schulte-Schrepping J, Reusch N, Paclik D, Bassler K, Schlickeiser S, Zhang B, Kramer B, Krammer T, Brumhard S, Bonaguro L (2020). Severe COVID-19 Is marked by a dysregulated myeloid cell compartment. Cell..

[17] Sundararaj Stanleyraj J, Sethuraman N, Gupta R, Thiruvoth S, Gupta M, Ryo A (2021). Treating COVID-19: are we missing out the window of opportunity?. J Antimicrob Chemother.

[18] Covid-19 Treatment guidelines. https://www.covid19treatmentguidelines.nih.gov/about-the-guidelines/whats-new/. Accessed July 7th, 2021

[19] Kim PS, Read SW, Fauci AS (2020). Therapy for early COVID-19: a critical need. JAMA.

[20] Cohen MS (2021). Monoclonal antibodies to disrupt progression of early Covid-19 infection. N Engl J Med.

[21] Meo SA, Bukhari IA, Akram J, Meo AS, Klonoff DC (2021). COVID-19 vaccines: comparison of biological, pharmacological characteristics and adverse effects of Pfizer/BioNTech and moderna vaccines. Eur Rev Med Pharmacol Sci.

[22] COVID-19 vaccines. In: Drugs and Lactation Database (LactMed)*.* Bethesda (MD); 2006.

[23] Poland GA, Ovsyannikova IG, Kennedy RB (2020). SARS-CoV-2 immunity: review and applications to phase 3 vaccine candidates. Lancet.

[24] Rosenbaum L (2021). Escaping Catch-22 - overcoming covid vaccine hesitancy. N Engl J Med.

[25] Fontanet A, Cauchemez S (2020). COVID-19 herd immunity: where are we?. Nat Rev Immunol.

[26] Kwok KO, Lai F, Wei WI, Wong SYS, Tang JWT (2020). Herd immunity - estimating the level required to halt the COVID-19 epidemics in affected countries. J Infect.

[27] Alaran AJ, Adebisi YA, Badmos A, Khalid-Salako F, Gaya SK, Ilesanmi EB, Olaoye DQ, Bamisaiye A, Lucero-Prisno DE (2021). 3^rd^. Uneven power dynamics must be levelled in COVID-19 vaccines access and distribution. Public Health Pract (Oxf)..

[28] Lucero-Prisno DE, Ogunkola IO, Imo UF, Adebisi YA. Who Will Pay for the COVID-19 Vaccines for Africa? Am J Trop Med Hyg*.* 2021. 10.4269/ajtmh.20-1506 PMC7941795 33427194

[29] Vaccine equity the ‘challenge of our time’, WHO chief declares, as governments call for solidarity, sharing. https://news.un.org/en/story/2021/04/1089972. Accessed July 7th, 2021

[30] COVAX: Working for global equitable access to covid-19 vaccines. https://www.who.int/initiatives/act-accelerator/covax. Accessed July 7th, 2021

[31] Sonani B, Aslam F, Goyal A, Patel J, Bansal P (2021). COVID-19 vaccination in immunocompromised patients. Clin Rheumatol.

[32] Coustasse A, Kimble C, Maxik K (2021). COVID-19 and vaccine hesitancy: a challenge the United States must overcome. J Ambul Care Manage.

[33] Group C (2020). A future vaccination campaign against COVID-19 at risk of vaccine hesitancy and politicisation. Lancet Infect Dis.

[34] Altmann DM, Boyton RJ, Beale R (2021). Immunity to SARS-CoV-2 variants of concern. Science.

[35] Abdool Karim SS, de Oliveira T: New SARS-CoV-2 Variants - Clinical, Public Health, and Vaccine Implications. N Engl J Med. 2021. 10.1056/NEJMc2100362 PMC8008749 33761203

[36] SARS-CoV-2 Variant Classifications and Definitions. .https://www.cdc.gov/coronavirus/2019-ncov/variants/variant-info.html. Accessed July 7th, 2021

[37] Tracking SARS-CoV-2 variants. https://www.who.int/en/activities/tracking-SARS-CoV-2-variants/. Accessed July 7th, 2021

[38] Khan A, Zia T, Suleman M, Khan T, Ali SS, Abbasi AA, Mohammad A, Wei DQ. Higher infectivity of the SARS-CoV-2 new variants is associated with K417N/T, E484K, and N501Y mutants: an insight from structural data. J Cell Physiol. 2021. 10.1002/jcp.30367 PMC8251074 33755190

[39] Gomez CE, Perdiguero B, Esteban M. Emerging SARS-CoV-2 Variants and impact in global vaccination programs against SARS-CoV-2/COVID-19. Vaccines (Basel). 2021, 9. 10.3390/vaccines9030243 PMC7999234 33799505

[40] Zhou D, Dejnirattisai W, Supasa P, Liu C, Mentzer AJ, Ginn HM, Zhao Y, Duyvesteyn HME, Tuekprakhon A, Nutalai R *et al*. Evidence of escape of SARS-CoV-2 variant B.1.351 from natural and vaccine-induced sera. Cell. 2021. 10.1016/j.cell.2021.02.037 PMC7901269 33730597

[41] Madhi SA, Baillie V, Cutland CL, Voysey M, Koen AL, Fairlie L, Padayachee SD, Dheda K, Barnabas SL, Bhorat QE *et al*. Efficacy of the ChAdOx1 nCoV-19 Covid-19 Vaccine against the B.1.351 Variant. N Engl J Med. 2021. 10.1056/NEJMoa2102214 PMC7993410 33725432

[42] Emary KRW, Golubchik T, Aley PK, Ariani CV, Angus B, Bibi S, Blane B, Bonsall D, Cicconi P, Charlton S *et al*. Efficacy of ChAdOx1 nCoV-19 (AZD1222) vaccine against SARS-CoV-2 variant of concern 202012/01 (B.1.1.7): an exploratory analysis of a randomised controlled trial. Lancet*.* 2021, **397**:1351–1362. 10.1016/S0140-6736(21)00628-0 PMC8009612 33798499

[43] Rubin R (2021). COVID-19 vaccines vs variants-determining how much immunity is enough. JAMA.

[44] Davis C, Logan N, Tyson G, Orton R, Harvey W, Haughney J, Perkins J, The COVID-19 genomics UK (COG-UK) consortium, peacock TP, Barclay WS et al. Reduced neutralisation of the Delta (B.1.617.2) SARS-CoV-2 variant of concern following vaccination. medRxiv 2021. 10.1371/journal.ppat.1010022 PMC8639073 34855916

[45] Sheikh A, McMenamin J, Taylor B, Robertson C, Public Health Scotland and the EAVE II Collaborators. SARS-CoV-2 Delta VOC in Scotland: demographics, risk of hospital admission, and vaccine effectiveness. Lancet. 2021, 397(10293):2461–2462. 10.1016/S0140-6736(21)01358-1 PMC8201647 34139198

[46] World Health Organization (WHO). COVID-19 weekly epidemiological update, 1 June 2021. Geneva: WHO; 2021. https://apps.who.int/iris/handle/10665/341622. Accessed July 7th, 2021

[47] Increased transmissibility and global spread of SARS-CoV-2 variants of concern as at June 2021. https://www.eurosurveillance.org/content/10.2807/1560-7917.ES.2021.26.24.2100509. Accessed July 7th, 2021 10.2807/1560-7917.ES.2021.26.24.2100509 PMC8212592 34142653

[48] Singh TU, Parida S, Lingaraju MC, Kesavan M, Kumar D, Singh RK (2020). Drug repurposing approach to fight COVID-19. Pharmacol Rep.

[49] Pawar AY. Combating devastating COVID-19 by drug repurposing. Int J Antimicrob Agents. 2020, 56:105984. 10.1016/j.ijantimicag.2020.105984 PMC7162749 32305589

[50] Consortium WHOST, Pan H, Peto R, Henao-Restrepo AM, Preziosi MP, Sathiyamoorthy V, Abdool Karim Q, Alejandria MM, Hernandez Garcia C, Kieny MP *et al*. Repurposed Antiviral Drugs for Covid-19 - Interim WHO solidarity trial results. N Engl J Med. 2021, 384:497–511. 10.1056/NEJMoa2023184 PMC7727327 33264556

[51] Del Rio C, Collins LF, Malani P. Long-term Health Consequences of COVID-19. JAMA. 2020. 10.1001/jama.2020.19719 PMC8019677 33031513

[52] Wrapp D, Wang N, Corbett KS, Goldsmith JA, Hsieh CL, Abiona O, Graham BS, McLellan JS (2020). Cryo-EM structure of the 2019-nCoV spike in the prefusion conformation. Science.

[53] Walls AC, Park YJ, Tortorici MA, Wall A, McGuire AT, Veesler D (2020). Structure, function, and antigenicity of the SARS-CoV-2 Spike glycoprotein. Cell.

[54] Li F (2016). Structure, function, and evolution of coronavirus spike proteins. Annu Rev Virol.

[55] Huang Y, Yang C, Xu XF, Xu W, Liu SW (2020). Structural and functional properties of SARS-CoV-2 spike protein: potential antivirus drug development for COVID-19. Acta Pharmacol Sin.

[56] Hoffmann M, Kleine-Weber H, Schroeder S, Kruger N, Herrler T, Erichsen S, Schiergens TS, Herrler G, Wu NH, Nitsche A (2020). SARS-CoV-2 cell entry depends on ACE2 and TMPRSS2 and is blocked by a clinically proven protease inhibitor. Cell..

[57] Martinez MA. Compounds with Therapeutic Potential against Novel Respiratory 2019 Coronavirus. Antimicrob Agents Chemother. 2020, 64(5). 10.1128/AAC.00399-20 PMC7179632 32152082

[58] Willis VC, Arriaga Y, Weeraratne D, Reyes F, Jackson GP (2020). A narrative review of emerging therapeutics for COVID-19. Mayo Clin Proc Innov Qual Outcomes.

[59] Peng HT, Rhind SG, Beckett A. Convalescent Plasma for the Prevention and Treatment of COVID-19: A systematic review and quantitative analysis. JMIR Public Health Surveill. 2021, 7:e25500. 10.2196/25500 PMC8245055 33825689

[60] Wooding DJ, Bach H (2020). Treatment of COVID-19 with convalescent plasma: lessons from past coronavirus outbreaks. Clin Microbiol Infect.

[61] Wang Y, Huo P, Dai R, Lv X, Yuan S, Zhang Y, Guo Y, Li R, Yu Q, Zhu K (2021). Convalescent plasma may be a possible treatment for COVID-19: A systematic review. Int Immunopharmacol..

[62] Renn A, Fu Y, Hu X, Hall MD, Simeonov A (2020). Fruitful neutralizing antibody pipeline brings hope to defeat SARS-Cov-2. Trends Pharmacol Sci.

[63] Jiang S, Zhang X, Yang Y, Hotez PJ, Du L (2020). Neutralizing antibodies for the treatment of COVID-19. Nat Biomed Eng.

[64] Seyedpour S, Khodaei B, Loghman AH, Seyedpour N, Kisomi MF, Balibegloo M, Nezamabadi SS, Gholami B, Saghazadeh A, Rezaei N (2021). Targeted therapy strategies against SARS-CoV-2 cell entry mechanisms: a systematic review of in vitro and in vivo studies. J Cell Physiol.

[65] Xiaojie S, Yu L, Lei Y, Guang Y, Min Q. Neutralizing antibodies targeting SARS-CoV-2 spike protein. Stem Cell Res. 2020, 50:102125. 10.1016/j.scr.2020.102125 PMC7737530 33341604

[66] Case JB, Winkler ES, Errico JM, Diamond MS (2021). On the road to ending the COVID-19 pandemic: are we there yet?. Virology.

[67] Weinreich DM, Sivapalasingam S, Norton T, Ali S, Gao H, Bhore R, Musser BJ, Soo Y, Rofail D, Im J (2021). REGN-COV2, a neutralizing antibody cocktail, in outpatients with Covid-19. N Engl J Med.

[68] Gottlieb RL, Nirula A, Chen P, Boscia J, Heller B, Morris J, Huhn G, Cardona J, Mocherla B, Stosor V (2021). Effect of bamlanivimab as monotherapy or in combination with etesevimab on viral load in patients with mild to moderate COVID-19: a randomized clinical trial. JAMA.

[69] EMERGENCY USE AUTHORIZATION (EUA) OF REGEN-COVTM (casirivimab with imdevimab). https://www.fda.gov/media/145611/download. Accessed July 7th, 2021

[70] Chen RE, Zhang X, Case JB, Winkler ES, Liu Y, VanBlargan LA, Liu J, Errico JM, Xie X, Suryadevara N *et al*. Resistance of SARS-CoV-2 variants to neutralization by monoclonal and serum-derived polyclonal antibodies. Nat Med. 2021. 10.1038/s41591-021-01294-w PMC8058618 33664494

[71] Kuzmina A, Khalaila Y, Voloshin O, Keren-Naus A, Boehm-Cohen L, Raviv Y, Shemer-Avni Y, Rosenberg E, Taube R (2021). SARS-CoV-2 spike variants exhibit differential infectivity and neutralization resistance to convalescent or post-vaccination sera. Cell Host Microbe..

[72] Wang P, Nair MS, Liu L, Iketani S, Luo Y, Guo Y, Wang M, Yu J, Zhang B, Kwong PD *et al*. Increased Resistance of SARS-CoV-2 Variants B.1.351 and B.1.1.7 to Antibody Neutralization. bioRxiv. 2021. 10.1038/s41586-021-03398-2 33684923

[73] Weisblum Y, Schmidt F, Zhang F, DaSilva J, Poston D, Lorenzi JC, Muecksch F, Rutkowska M, Hoffmann HH, Michailidis E *et al*. Escape from neutralizing antibodies by SARS-CoV-2 spike protein variants. Elife. 2020, 9. 10.7554/eLife.61312 PMC7723407 33112236

[74] Hoffmann M, Hofmann-Winkler H, Krüger N, Kempf A, Nehlmeier I, Graichen L, Sidarovich A, Moldenhauer AS, Winkler MS, Schulz S et al. SARS-CoV-2 variant B.1.617 is resistant to Bamlanivimab and evades antibodies induced by infection and vaccination. bioRxiv, 2021. 10.1016/j.celrep.2021.109415 PMC8238662 34270919

[75] Bamlanivimab. https://www.phe.gov/emergency/events/COVID19/investigation-MCM/Bamlanivimab/Pages/default.aspx. Accessed July 7^th^, 2021

[76] EMERGENCY USE AUTHORIZATION (EUA) OF BAMLANIVIMAB AND ETESEVIMAB. https://www.fda.gov/media/145802/download. Accessed July 7thth, 2021

[77] Tada T, Zhou H, Dcostaa BM, Samanovicb MI, Mulliganb MJ, Landaua NR. The Spike Proteins of SARS-CoV-2 B.1.617 and B.1.618 Variants Identified in India Provide Partial Resistance to Vaccine-elicited and Therapeutic Monoclonal Antibodies. bioRxiv, 2021.

[78] Rappazzo CG, Tse LV, Kaku CI, Wrapp D, Sakharkar M, Huang D, Deveau LM, Yockachonis TJ, Herbert AS, Battles MB (2021). Broad and potent activity against SARS-like viruses by an engineered human monoclonal antibody. Science.

[79] Wec AZ, Wrapp D, Herbert AS, Maurer DP, Haslwanter D, Sakharkar M, Jangra RK, Dieterle ME, Lilov A, Huang D (2020). Broad neutralization of SARS-related viruses by human monoclonal antibodies. Science.

[80] Starr TN, Czudnochowski N, Zatta F, Park YJ, Liu Z, Addetia A, Pinto D, Beltramello M, Hernandez P, Greaney AJ *et al*. Antibodies to the SARS-CoV-2 receptor-binding domain that maximize breadth and resistance to viral escape. bioRxiv*.* 2021. 10.1038/s41586-021-03807-6 PMC9282883 34261126

[81] Tortorici MA, Czudnochowski N, Starr TN, Marzi R, Walls AC, Zatta F, Bowen JE, Jaconi S, Iulio JD, Wang Z *et al*. Structural basis for broad sarbecovirus neutralization by a human monoclonal antibody. bioRxiv. 2021. 10.1038/s41586-021-03817-4 PMC9341430 34280951

[82] Baum A, Fulton BO, Wloga E, Copin R, Pascal KE, Russo V, Giordano S, Lanza K, Negron N, Ni M (2020). Antibody cocktail to SARS-CoV-2 spike protein prevents rapid mutational escape seen with individual antibodies. Science.

[83] Pinto D, Park YJ, Beltramello M, Walls AC, Tortorici MA, Bianchi S, Jaconi S, Culap K, Zatta F, De Marco A (2020). Cross-neutralization of SARS-CoV-2 by a human monoclonal SARS-CoV antibody. Nature.

[84] Liu H, Yuan M, Huang D, Bangaru S, Zhao F, Lee CD, Peng L, Barman S, Zhu X, Nemazee D et al. A combination of cross-neutralizing antibodies synergizes to prevent SARS-CoV-2 and SARS-CoV pseudovirus infection. Cell Host Microbe. 2021. 10.1016/j.chom.2021.04.005 PMC8049401 33894127

[85] Sasisekharan R. **P**reparing for the Future - Nanobodies for Covid-19? N Engl J Med. 2021. 10.1056/NEJMcibr2101205 33826817

[86] Konwarh R: Nanobodies Prospects of expanding the gamut of neutralizing antibodies against the novel coronavirus, SARS-CoV-2. Front Immunol. 2020, 11:1531. 10.3389/fimmu.2020.01531 PMC7324746 32655584

[87] Huo J, Le Bas A, Ruza RR, Duyvesteyn HME, Mikolajek H, Malinauskas T, Tan TK, Rijal P, Dumoux M, Ward PN (2020). Neutralizing nanobodies bind SARS-CoV-2 spike RBD and block interaction with ACE2. Nat Struct Mol Biol.

[88] Chi X, Liu X, Wang C, Zhang X, Li X, Hou J, Ren L, Jin Q, Wang J, Yang W (2020). Humanized single domain antibodies neutralize SARS-CoV-2 by targeting the spike receptor binding domain. Nat Commun.

[89] Koenig PA, Das H, Liu H, Kummerer BM, Gohr FN, Jenster LM, Schiffelers LDJ, Tesfamariam YM, Uchima M, Wuerth JD *et al*. Structure-guided multivalent nanobodies block SARS-CoV-2 infection and suppress mutational escape. Science*.* 2021, 371. 10.1126/science.abe6230 PMC7932109 33436526

[90] Xiang Y, Nambulli S, Xiao Z, Liu H, Sang Z, Duprex WP, Schneidman-Duhovny D, Zhang C, Shi Y (2020). Versatile and multivalent nanobodies efficiently neutralize SARS-CoV-2. Science.

[91] Xu J, Xu K, Jung S, Conte A, Lieberman J, Muecksch F, Cetrulo Lorenzi JC, Park S, Wang Z, Tessarollo L *et al*. Multimeric nanobodies from camelid engineered mice and llamas potently neutralize SARS-CoV-2 variants. bioRxiv*.* 2021. 10.1038/s41586-021-03676-z PMC8260353 34098567

[92] Schoof M, Faust B, Saunders RA, Sangwan S, Rezelj V, Hoppe N, Boone M, Billesbolle CB, Puchades C, Azumaya CM (2020). An ultrapotent synthetic nanobody neutralizes SARS-CoV-2 by stabilizing inactive Spike. Science.

[93] Bracken CJ, Lim SA, Solomon P, Rettko NJ, Nguyen DP, Zha BS, Schaefer K, Byrnes JR, Zhou J, Lui I (2021). Bi-paratopic and multivalent VH domains block ACE2 binding and neutralize SARS-CoV-2. Nat Chem Biol.

[94] Sun D, Sang Z, Kim YJ, Xiang Y, Cohen T, Belford AK, Huet A, Conway JF, Sun J, Taylor DJ *et al*. Potent neutralizing nanobodies resist convergent circulating variants of SARS-CoV-2 by targeting novel and conserved epitopes. bioRxiv*.* 2021. 10.1038/s41467-021-24963-3 PMC8333356 34344900

[95] Mast FD, Fridy PC, Ketaren NE, Wang J, Jacobs EY, Olivier JP, Sanyal T, Molloy KR, Schmidt F, Rutkowska M *et al*. Nanobody repertoires for exposing vulnerabilities of SARS-CoV-2. bioRxiv. 2021.

[96] Pymm P, Adair A, Chan LJ, Cooney JP, Mordant FL, Allison CC, Lopez E, Haycroft ER, O'Neill MT, Tan LL *et al*. Nanobody cocktails potently neutralize SARS-CoV-2 D614G N501Y variant and protect mice**.** Proc Natl Acad Sci USA. 2021, 118. 10.1073/pnas.2101918118 PMC8126837 33893175

[97] Baum A, Ajithdoss D, Copin R, Zhou A, Lanza K, Negron N, Ni M, Wei Y, Mohammadi K, Musser B (2020). REGN-COV2 antibodies prevent and treat SARS-CoV-2 infection in rhesus macaques and hamsters. Science.

[98] Rosenfeld R, Noy-Porat T, Mechaly A, Makdasi E, Levy Y, Alcalay R, Falach R, Aftalion M, Epstein E, Gur D (2021). Post-exposure protection of SARS-CoV-2 lethal infected K18-hACE2 transgenic mice by neutralizing human monoclonal antibody. Nat Commun.

[99] Kreye J, Reincke SM, Kornau HC, Sanchez-Sendin E, Corman VM, Liu H, Yuan M, Wu NC, Zhu X, Lee CD *et al*. A Therapeutic Non-self-reactive SARS-CoV-2 Antibody Protects from Lung Pathology in a COVID-19 Hamster Model. Cell*.* 2020, 183:1058–1069 e1019. 10.1016/j.cell.2020.09.049 PMC7510528 33058755

[100] Li W, Chen C, Drelich A, Martinez DR, Gralinski LE, Sun Z, Schafer A, Kulkarni SS, Liu X, Leist SR (2020). Rapid identification of a human antibody with high prophylactic and therapeutic efficacy in three animal models of SARS-CoV-2 infection. Proc Natl Acad Sci U S A.

[101] Winkler ES, Gilchuk P, Yu J, Bailey AL, Chen RE, Chong Z, Zost SJ, Jang H, Huang Y, Allen JD (2021). Human neutralizing antibodies against SARS-CoV-2 require intact Fc effector functions for optimal therapeutic protection. Cell..

[102] Phase III Double-blind, Placebo-controlled Study of AZD7442 for Pre-exposure Prophylaxis of COVID-19 in Adult.

[103] COVID-19 Study Assessing the Efficacy and Safety of Anti-Spike SARS CoV-2 Monoclonal Antibodies for Prevention of SARS CoV-2 Infection Asymptomatic in Healthy Adults and Adolescents Who Are Household Contacts to an Individual With a Positive SARS-CoV-2 RT-PCR Assay.

[104] A Study of LY3819253 (LY-CoV555) and LY3832479 (LY-CoV016) in Preventing SARS-CoV-2 Infection and COVID-19 in Nursing Home Residents and Staff (BLAZE-2).

[105] Monteil V, Kwon H, Prado P, Hagelkruys A, Wimmer RA, Stahl M, Leopoldi A, Garreta E, Hurtado Del Pozo C, Prosper F (2020). Inhibition of SARS-CoV-2 infections in engineered human tissues using clinical-grade soluble human ACE2. Cell..

[106] Zoufaly A, Poglitsch M, Aberle JH, Hoepler W, Seitz T, Traugott M, Grieb A, Pawelka E, Laferl H, Wenisch C (2020). Human recombinant soluble ACE2 in severe COVID-19. Lancet Respir Med.

[107] Recombinant Human Angiotensin-converting Enzyme 2 (rhACE2) as a Treatment for Patients With COVID-19 (APN01-COVID-19).

[108] Chan KK, Tan TJC, Narayanan KK, Procko E. An engineered decoy receptor for SARS-CoV-2 broadly binds protein S sequence variants. Sci Adv*.* 2021, 7. 10.1126/sciadv.abf1738 PMC7888922 33597251

[109] Glasgow A, Glasgow J, Limonta D, Solomon P, Lui I, Zhang Y, Nix MA, Rettko NJ, Zha S, Yamin R (2020). Engineered ACE2 receptor traps potently neutralize SARS-CoV-2. Proc Natl Acad Sci U S A.

[110] Linsky TW, Vergara R, Codina N, Nelson JW, Walker MJ, Su W, Barnes CO, Hsiang TY, Esser-Nobis K, Yu K (2020). De novo design of potent and resilient hACE2 decoys to neutralize SARS-CoV-2. Science.

[111] Pomplun S (2021). Targeting the SARS-CoV-2-spike protein: from antibodies to miniproteins and peptides. RSC Med Chem.

[112] Cao L, Goreshnik I, Coventry B, Case JB, Miller L, Kozodoy L, Chen RE, Carter L, Walls AC, Park YJ (2020). De novo design of picomolar SARS-CoV-2 miniprotein inhibitors. Science.

[113] Schutz D, Ruiz-Blanco YB, Munch J, Kirchhoff F, Sanchez-Garcia E, Muller JA (2020). Peptide and peptide-based inhibitors of SARS-CoV-2 entry. Adv Drug Deliv Rev.

[114] Han DP, Penn-Nicholson A, Cho MW (2006). Identification of critical determinants on ACE2 for SARS-CoV entry and development of a potent entry inhibitor. Virology.

[115] Zheng BJ, Guan Y, Hez ML, Sun H, Du L, Zheng Y, Wong KL, Chen H, Chen Y, Lu L (2005). Synthetic peptides outside the spike protein heptad repeat regions as potent inhibitors of SARS-associated coronavirus. Antivir Ther.

[116] Ho TY, Wu SL, Chen JC, Wei YC, Cheng SE, Chang YH, Liu HJ, Hsiang CY (2006). Design and biological activities of novel inhibitory peptides for SARS-CoV spike protein and angiotensin-converting enzyme 2 interaction. Antiviral Res.

[117] Hu H, Li L, Kao RY, Kou B, Wang Z, Zhang L, Zhang H, Hao Z, Tsui WH, Ni A (2005). Screening and identification of linear B-cell epitopes and entry-blocking peptide of severe acute respiratory syndrome (SARS)-associated coronavirus using synthetic overlapping peptide library. J Comb Chem.

[118] Karoyan P, Vieillard V, Gomez-Morales L, Odile E, Guihot A, Luyt CE, Denis A, Grondin P, Lequin O (2021). Human ACE2 peptide-mimics block SARS-CoV-2 pulmonary cells infection. Commun Biol.

[119] Curreli F, Victor SMB, Ahmed S, Drelich A, Tong X, Tseng CK, Hillyer CD, Debnath AK. Stapled Peptides Based on Human Angiotensin-Converting Enzyme 2 (ACE2) Potently Inhibit SARS-CoV-2 Infection In Vitro. mBio*.* 2020, 11. 10.1128/mBio.02451-20 PMC7751257 33310780

[120] Watson A, Ferreira, L MR, Hwang P, Xu J, Stroud R. Peptide antidotes to SARS-CoV-2 (COVID-19). BioRxiv. 2020.

[121] Zhang G,Pomplun S, Loftis AR, Loas A, Pentelute BL. The first-in-class peptide binder to the SARS-CoV-2 spike protein. bioRxiv. 2020

[122] Zhang G,Pomplun S, Loftis AR, Tan X, Loas A, Pentelute BL. Investigation of ACE2 N-terminal fragments binding to SARS- CoV-2 Spike RBD. bioRxiv. 2020

[123] Morgan DC, Morris C, Mahindra A, Blair CM, Tejeda G, Herbert I, Turnbull ML, Lieber G, Willett BJ, Logan N *et al*. Stapled ACE2 peptidomimetics designed to target the SARS-CoV-2 spike protein do not prevent virus internalization. Pept Sci (Hoboken). 2021:e24217. 10.1002/pep2.24217 PMC7883042 33615115

[124] Xia S, Xu W, Wang Q, Wang C, Hua C, Li W, Lu L, Jiang S. Peptide-Based Membrane Fusion Inhibitors Targeting HCoV-229E Spike Protein HR1 and HR2 Domains. Int J Mol Sci. 2018, 19. 10.3390/ijms19020487 PMC5855709 29415501

[125] Bosch BJ, Martina BE, Van Der Zee R, Lepault J, Haijema BJ, Versluis C, Heck AJ, De Groot R, Osterhaus AD, Rottier PJ (2004). Severe acute respiratory syndrome coronavirus (SARS-CoV) infection inhibition using spike protein heptad repeat-derived peptides. Proc Natl Acad Sci U S A.

[126] Sun Y, Zhang H, Shi J, Zhang Z, Gong R. Identification of a Novel Inhibitor against Middle East Respiratory Syndrome Coronavirus. Viruses. 2017, 9. 10.3390/v9090255 PMC5618021 28906430

[127] Xia S, Yan L, Xu W, Agrawal AS, Algaissi A, Tseng CK, Wang Q, Du L, Tan W, Wilson IA *et al*. A pan-coronavirus fusion inhibitor targeting the HR1 domain of human coronavirus spike. Sci Adv. 2019, 5:eaav4580. 10.1126/sciadv.aav4580 PMC6457931 30989115

[128] Xia S, Liu M, Wang C, Xu W, Lan Q, Feng S, Qi F, Bao L, Du L, Liu S (2020). Inhibition of SARS-CoV-2 (previously 2019-nCoV) infection by a highly potent pan-coronavirus fusion inhibitor targeting its spike protein that harbors a high capacity to mediate membrane fusion. Cell Res.

[129] Zhu Y, Yu D, Yan H, Chong H, He Y. Design of Potent Membrane Fusion Inhibitors against SARS-CoV-2, an Emerging Coronavirus with High Fusogenic Activity. J Virol. 2020, 94. 10.1128/JVI.00635-20 PMC7343218 32376627

[130] de Vries RD, Schmitz KS, Bovier FT, Predella C, Khao J, Noack D, Haagmans BL, Herfst S, Stearns KN, Drew-Bear J (2021). Intranasal fusion inhibitory lipopeptide prevents direct-contact SARS-CoV-2 transmission in ferrets. Science.

[131] Sanders JM, Monogue ML, Jodlowski TZ, Cutrell JB (2020). Pharmacologic treatments for coronavirus disease 2019 (COVID-19): a review. JAMA.

[132] Xiu S, Dick A, Ju H, Mirzaie S, Abdi F, Cocklin S, Zhan P, Liu X (2020). Inhibitors of SARS-CoV-2 entry: current and future opportunities. J Med Chem.

[133] Khare P, Sahu U, Pandey SC, Samant M (2020). Current approaches for target-specific drug discovery using natural compounds against SARS-CoV-2 infection. Virus Res..

[134] Artese A, Svicher V, Costa G, Salpini R, Di Maio VC, Alkhatib M, Ambrosio FA, Santoro MM, Assaraf YG, Alcaro S (2020). Current status of antivirals and druggable targets of SARS CoV-2 and other human pathogenic coronaviruses. Drug Resist Updat..

[135] Jan JT, Cheng TR, Juang YP, Ma HH, Wu YT, Yang WB, Cheng CW, Chen X, Chou TH, Shie JJ *et al*. Identification of existing pharmaceuticals and herbal medicines as inhibitors of SARS-CoV-2 infection. Proc Natl Acad Sci U S A. 2021, 118. 10.1073/pnas.2021579118 PMC7865145 33452205

[136] Ohashi H, Watashi K, Saso W, Shionoya K, Iwanami S, Hirokawa T, Shirai T, Kanaya S, Ito Y, Kim KS *et al*. Potential anti-COVID-19 agents, cepharanthine and nelfinavir, and their usage for combination treatment. iScience. 2021, 24:102367. 10.1016/j.isci.2021.102367 PMC7997640 33817567

[137] Dittmar M, Lee JS, Whig K, Segrist E, Li M, Kamalia B, Castellana L, Ayyanathan K, Cardenas-Diaz FL, Morrisey EE (2021). Drug repurposing screens reveal cell-type-specific entry pathways and FDA-approved drugs active against SARS-Cov-2. Cell Rep..

[138] Jeon S, Ko M, Lee J, Choi I, Byun SY, Park S, Shum D, Kim S. Identification of antiviral drug candidates against SARS-CoV-2 from FDA-approved drugs. Antimicrob Agents Chemother. 2020, 64. 10.1128/AAC.00819-20 PMC7318052 32366720

[139] Ko M, Jeon S, Ryu WS, Kim S (2021). Comparative analysis of antiviral efficacy of FDA-approved drugs against SARS-CoV-2 in human lung cells. J Med Virol.

[140] Riva L, Yuan S, Yin X, Martin-Sancho L, Matsunaga N, Pache L, Burgstaller-Muehlbacher S, De Jesus PD, Teriete P, Hull MV (2020). Discovery of SARS-CoV-2 antiviral drugs through large-scale compound repurposing. Nature.

[141] He CL, Huang LY, Wang K, Gu CJ, Hu J, Zhang GJ, Xu W, Xie YH, Tang N, Huang AL (2021). Identification of bis-benzylisoquinoline alkaloids as SARS-CoV-2 entry inhibitors from a library of natural products. Signal Transduct Target Ther.

[142] Hanson QM, Wilson KM, Shen M, Itkin Z, Eastman RT, Shinn P, Hall MD (2020). Targeting ACE2-RBD interaction as a platform for COVID-19 therapeutics: development and drug-repurposing screen of an alphalisa proximity assay. ACS Pharmacol Transl Sci.

[143] Day CJ, Bailly B, Guillon P, Dirr L, Jen FE, Spillings BL, Mak J, von Itzstein M, Haselhorst T, Jennings MP. Multidisciplinary Approaches Identify Compounds that Bind to Human ACE2 or SARS-CoV-2 Spike Protein as Candidates to Block SARS-CoV-2-ACE2 Receptor Interactions. mBio*.* 2021, 12. 10.1128/mBio.03681-20 PMC8092326 33785634

[144] Nabavi SF, Habtemariam S, Berindan-Neagoe I, Cismaru CA, Schaafsma D, Ghavami S, Banach M, Aghaabdollahian S, Nabavi SM (2021). Rationale for effective prophylaxis against COVID-19 through simultaneous blockade of both endosomal and non-endosomal SARS-CoV-2 Entry into Host Cell. Clin Transl Sci.

[145] Cannalire R, Stefanelli I, Cerchia C, Beccari AR, Pelliccia S, Summa V. SARS-CoV-2 Entry Inhibitors: Small Molecules and Peptides Targeting Virus or Host Cells. Int J Mol Sci*.* 2020, 21. 10.3390/ijms21165707 PMC7460888 32784899

[146] Bestle D, Heindl MR, Limburg H, Van Lam van T, Pilgram O, Moulton H, Stein DA, Hardes K, Eickmann M, Dolnik O *et al*. TMPRSS2 and furin are both essential for proteolytic activation of SARS-CoV-2 in human airway cells. Life Sci Alliance. 2020, 3. 10.26508/lsa.202000786 PMC7383062 32703818

[147] Hoffmann M, Kleine-Weber H, Pohlmann S (2020). A multibasic cleavage site in the spike protein of SARS-CoV-2 is essential for infection of human lung cells. Mol Cell..

[148] Böttcher-Friebertshäuser E. Membrane-anchored serine proteases: host cell factors in proteolytic activation of viral glycoproteins. Activation of Viruses by Host Proteases. 2018 :153–203.

[149] Zhirnov OP, Klenk HD, Wright PF (2011). Aprotinin and similar protease inhibitors as drugs against influenza. Antiviral Res.

[150] Yamamoto M, Kiso M, Sakai-Tagawa Y, Iwatsuki-Horimoto K, Imai M, Takeda M, Kinoshita N, Ohmagari N, Gohda J, Semba K *et al*. The Anticoagulant Nafamostat Potently Inhibits SARS-CoV-2 S Protein-Mediated Fusion in a Cell Fusion Assay System and Viral Infection In Vitro in a Cell-Type-Dependent Manner. Viruses. 2020, 12. 10.3390/v12060629 PMC7354595 32532094

[151] Hoffmann M, Schroeder S, Kleine-Weber H, Muller MA, Drosten C, Pohlmann S. Nafamostat mesylate blocks activation of SARS-CoV-2: new treatment option for COVID-19. Antimicrob Agents Chemother*.* 2020, 64. 10.1128/AAC.00754-20 PMC7269515 32312781

[152] Yang N, Shen HM (2020). Targeting the endocytic pathway and autophagy process as a novel therapeutic strategy in COVID-19. Int J Biol Sci.

[153] Das G, Ghosh S, Garg S, Ghosh S, Jana A, Samat R, Mukherjee N, Roya R, Ghosh S (2020). An overview of key potential therapeutic strategies for combat in the covid-19 battle. RSC Adv.

[154] Zhang J, Ma X, Yu F, Liu J, Zou F, Pan T, Zhang H. **T**eicoplanin potently blocks the cell entry of 2019‐nCoV. bioRxiv. 2020.

[155] Ou X, Liu Y, Lei X, Li P, Mi D, Ren L, Guo L, Guo R, Chen T, Hu J (2020). Characterization of spike glycoprotein of SARS-CoV-2 on virus entry and its immune cross-reactivity with SARS-CoV. Nat Commun.

[156] Zhao H, To KKW, Sze KH, Yung TT, Bian M, Lam H, Yeung ML, Li C, Chu H, Yuen KY (2020). A broad-spectrum virus- and host-targeting peptide against respiratory viruses including influenza virus and SARS-CoV-2. Nat Commun.

[157] Zhao H, To KKW, Lam H, Zhou X, Chan JF, Peng Z, Lee ACY, Cai J, Chan WM, Ip JD (2021). Cross-linking peptide and repurposed drugs inhibit both entry pathways of SARS-CoV-2. Nat Commun.

[158] Andreani J, Le Bideau M, Duflot I, Jardot P, Rolland C, Boxberger M, Wurtz N, Rolain JM, Colson P, La Scola B *et al*. In vitro testing of combined hydroxychloroquine and azithromycin on SARS-CoV-2 shows synergistic effect. Microb Pathog. 2020, 145:104228. 10.1016/j.micpath.2020.104228 PMC7182748 32344177

[159] Haydar D, Cory TJ, Birket SE, Murphy BS, Pennypacker KR, Sinai AP, Feola DJ (2019). Azithromycin polarizes macrophages to an M2 phenotype via inhibition of the STAT1 and NF-kappaB signaling pathways. J Immunol.

[160] Nujic K, Banjanac M, Munic V, Polancec D, Erakovic Haber V (2012). Impairment of lysosomal functions by azithromycin and chloroquine contributes to anti-inflammatory phenotype. Cell Immunol.

[161] Shang C, Zhuang X, Zhang H, Li Y, Zhu Y, Lu J, Ge C, Cong J, Li T, Tian M (2021). Inhibitors of endosomal acidification suppress SARS-CoV-2 replication and relieve viral pneumonia in hACE2 transgenic mice. Virol J.

[162] Galan LEB, Santos NMD, Asato MS, Araujo JV, de Lima Moreira A, Araujo AMM, Paiva ADP, Portella DGS, Marques FSS, Silva GMA *et al*. Phase 2 randomized study on chloroquine, hydroxychloroquine or ivermectin in hospitalized patients with severe manifestations of SARS-CoV-2 infection. Pathog Glob Health. 2021:1–8. 10.1080/20477724.2021.1890887 PMC7938655 33682640

[163] Cavalcanti AB, Zampieri FG, Rosa RG, Azevedo LCP, Veiga VC, Avezum A, Damiani LP, Marcadenti A, Kawano-Dourado L, Lisboa T (2020). Hydroxychloroquine with or without Azithromycin in Mild-to-Moderate Covid-19. N Engl J Med.

[164] Shagufta Ahmad I (2021). The race to treat COVID-19: Potential therapeutic agents for the prevention and treatment of SARS-CoV-2. Eur J Med Chem.

[165] Stamatatos L, Czartoski J, Wan YH, Homad LJ, Rubin V, Glantz H, Neradilek M, Seydoux E, Jennewein MF, MacCamy AJ et al, mRNA vaccination boosts cross-variant neutralizing antibodies elicited by SARS-CoV-2 infection. Science. 2021, eabg9175. 10.1126/science.abg9175 PMC8139425 33766944

[166] Lopez Bernal J, Andrews N, Gower C, Gallagher E, Utsi L, Simmons R, Thelwall S, Stowe J, Tessier E, Groves N, Dabrera G et al. Effectiveness of COVID-19 vaccines against the B.1.617.2 (Delta) variant. bioRxiv, 2021. 10.1056/NEJMoa2108891 PMC8314739 34289274

[167] Julia Stowe , Nick Andrews, Charlotte Gower , Eileen Gallagher, Lara Utsi , Ruth Simmons, Simon Thelwall, Elise Tessier, Natalie Groves, Gavin Dabrera et al. Effectiveness of COVID-19 vaccines against hospital admission with the Delta (B.1.617.2) variant. PHE Preprint, 2021. 10.1056/NEJMoa2108891 PMC8314739 34289274

[168] Katella K. Comparing the COVID-19 Vaccines: How Are They Different? https://www.yalemedicine.org/news/covid-19-vaccine-comparison. Accessed July 7th, 2021

[169] Meganck RM, Baric RS (2021). Developing therapeutic approaches for twenty-first-century emerging infectious viral diseases. Nat Med.

[170] National security directive united states global leadership to strengthen the international covid-19 response and to advance global health security and biological preparedness. https://www.whitehouse.gov/briefing-room/statements-releases/2021/01/21/national-security-directive-united-states-global-leadership-to-strengthen-the-international-covid-19-response-and-to-advance-global-health-security-and-biological-preparedness/. Accessed July 7th, 2021.

[171] Global leaders unite in urgent call for international pandemic treaty. https://www.who.int/news/item/30-03-2021-global-leaders-unite-in-urgent-call-for-international-pandemic-treaty. Accessed July 7th^,^ 2021.

